# Geographic origin, ancestry, and death circumstances at the Cornaux/Les Sauges Iron Age bridge, Switzerland

**DOI:** 10.1038/s41598-024-62524-y

**Published:** 2024-06-17

**Authors:** Zita Laffranchi, Stefania Zingale, Lara Indra, Valentina Coia, Domingo C. Salazar García, Alice Paladin, Marc-Antoine Kaeser, Géraldine Delley, Sönke Szidat, Sandra Lösch, Albert Zink, Marco Milella

**Affiliations:** 1 Department of Physical Anthropology, Institute of Forensic Medicine, University of Bern, Bern, Switzerland; 2 Institute for Mummy Studies, Eurac Research, Bolzano, Italy; 3 Departament de Prehistòria, Arqueologia i Història Antiga, Universitat de València, Valencia, Spain; 4 Department of Geological Sciences, University of Cape Town, Cape Town, South Africa; 5 Laténium, Parc et Musée d’archéologie, Neuchâtel, Switzerland; 6 Department of Chemistry, Biochemistry and Pharmaceutical Sciences and Oeschger Centre for Climate Change Research, University of Bern, Bern, Switzerland; 7 Ludwig. Maximilians- Universität München, Munich, Germany

**Keywords:** Biogeochemistry, Genetics, Anthropology, Archaeology, Biological anthropology, Social anthropology

## Abstract

Cornaux/Les Sauges (Switzerland, Late Iron Age) revealed remnants of a wooden bridge, artifacts, and human and animal skeletal remains. The relationship between the collapsed structure and the skeletal material, whether it indicates a potential accident or cultural practices, remains elusive. We evaluate the most plausible scenario for Cornaux based on osteological, taphonomic, isotopic, and paleogenomic analysis of the recovered individuals. The latter amount to at least 20 individuals, mostly adult males. Perimortem lesions include only blunt force traumas. Radiocarbon data fall between the 3rd and 1st c. BCE, although in some cases predating available dendrochronological estimates from the bridge. Isotopic data highlight five to eight nonlocals. No close genetic relatedness links the analyzed skeletons. Paleogenomic results, the first for Iron Age Switzerland, point to a genetic affinity with other Central and Western European Iron Age groups. The type of skeletal lesions supports an accidental event as the more plausible explanation. Radiocarbon data and the demographic structure of the sample may suggest a sequence of different events possibly including executions and/or sacrifices. Isotopic and paleogenomic data, while not favoring one scenario over the other, do support earlier interpretations of the last centuries BCE in Europe as a dynamic period from a biocultural perspective.

## Introduction

**Figure 1:**
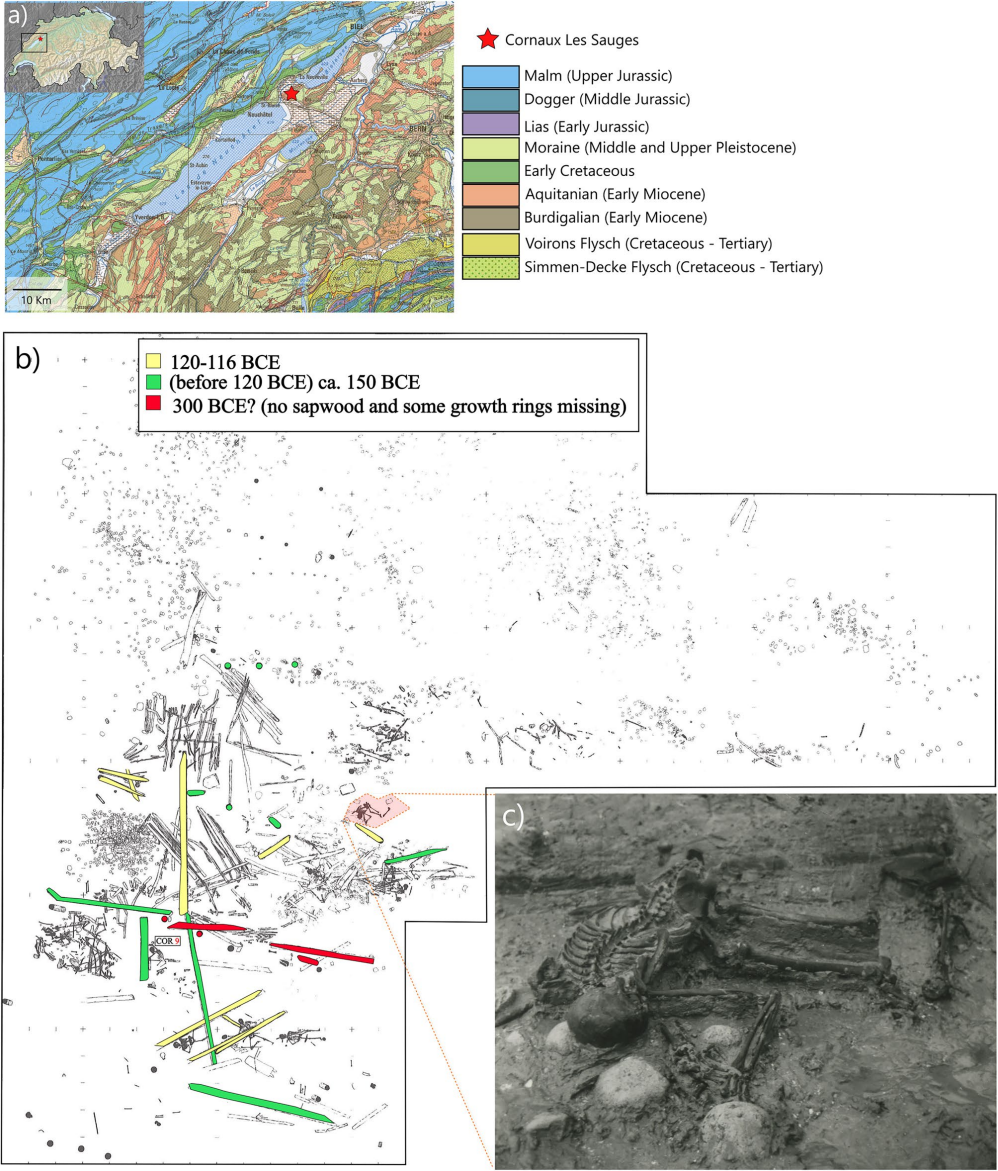
Cornaux/Les Sauges: geographic position and archaeological plan. (**a**) geographic position of Cornaux/Les Sauges and simplified geological map of the surrounding areas; (**b**) archaeological plan of the site with colors indicating the dendrochronological dates for some of the wood beams, and (**c**) picture of COR-16 in situ. Geological map modified from swisstopo (https://www.swisstopo.admin.ch/). Archaeological plan and excavation pictures courtesy of Laténium—Archaeology Park and Museum Neuchâtel.

The Late Iron Age (5th–1st c. BCE) in Europe is well known for distinctive depositions of weapons that are sometimes accompanied by various utensils and animal and/or human remains. In some cases, the deposited objects (e.g., weapons), their intentional damage, and the specific placement suggest the ritual nature of these assemblages. Examples of this type of find include those from Gournay-sur-Aronde^1^ and Le Cailar^2^ in France, Hayling Island^3^ in England, and Tiefenau^4^, Port^5^, and La Tène^5–8^ in Switzerland. In some instances, the processes and the actual meaning of these depositions are more elusive, with one particularly puzzling example being Cornaux/Les Sauges (henceforth Cornaux), in the canton of Neuchâtel, Western Switzerland (Fig. 1a). This site continues to attract the interest of the scientific community due to its unique combination of archaeological and anthropological features. Discovered and excavated between 1965 and 1966, the site covers a surface of 750 m^2^ on the banks of the river Thielle, 3 km from the eponymous site of La Tène (see Supplementary Text). The archaeological works revealed architectural timbers chaotically distributed in the riverbed and having belonged to a bridge built according to techniques attested in the region for the Iron Age and Roman period^9,10^. Dendrochronological data^11,12^ pointed to ca. 135 BCE for the construction of the bridge, with partial repairs in 120–115 and 105 BCE. The typology of pottery and iron and bronze items, including weapons (e.g., swords and spearheads), from the site mostly corresponds with these dates, thus falling in the La Tène D1 phase (150–90 BCE) with only few elements (i.e., a possible settlement on the upper part of the slope) suggesting a prior phase (La Tène C), and a later chronology (La Tène D2). In the original publication^13^, the association between the skeletons and the archaeological finds, particularly the weapons, was assumed without considering taphonomic factors. Although the site also yields numerous faunal remains, the most intriguing discovery was that of ca. 20 human individuals, represented by partial or whole skeletons, in some cases entangled with the beams of the bridge deck and framework^13^ (Fig. 1b,c). In five cases, the recovery of soft tissue remains from the endocranium attests to a partial preservation of brain tissue^14,15^. The animal skeletal remains (of three cattle and two horses) were recovered on both the lower bank and the river bed alongside most of the human skeletons and had some preserved anatomical connections, with few instances of cutting or butchering marks and none of which are clearly related to sacrificial (or intentional) actions^12,13^.

Several anthropological and archaeological features complicate the discussion surrounding this site's interpretation. First, its similarity to Late Iron Age ritual contexts featuring the apparent deposition of weaponry, objects, humans, and/or animals in water environments^16–21^. In connection to this, excavations at Cornaux recovered an intentionally folded bronze sheet of a sword scabbard, indicating a connection to the ritual destruction of weapons attested in the “Celtic” world in the 3rd and 2nd c. BCE^22^. Second, the results of previous anthropological surveys^23,24^ highlighted a majority of adult males among the recovered skeletons as well as the presence of traumatic lesions of an unclarified nature (intentional vs. accidental). Third, the position of the skeletons, in some cases “pinned” under the wood beans and presenting heterogeneous orientation and anatomical position.

The combination of these features has led to the development of two alternative interpretive hypotheses for this site, such as: (i) the collapse of the bridge, possibly related to a natural event, leading to the dispersal of people and animals crossing this structure^12,13,25–28^, (ii) the ritual offerings of humans, animals, and objects, as documented archaeologically for the Late Iron Age in both continental Europe and Britain^4,21,29–31^.

Almost 60 years after their discovery, our study arises from the need to re-evaluate the anthropological finds from Cornaux using a multidisciplinary set of analytical methods including osteological, isotopic, and ancient DNA analyses. We want to emphasize that the objective of this study is *not* to provide a definitive answer to the dynamics that occurred at the site, but rather evaluate which scenario is better supported by the data emerged from these different analyses.

## Results

### Osteological and taphonomic analysis

The demographic structure of the human sample (number of individuals, sex and age-at-death distribution), along with the potential presence of traumatic lesions on the skeletons, may provide valuable insights when evaluating the different interpretive models for Cornaux. At least 20 individuals (MNI) were calculated based on the left femur (both proximal and middle third of the femoral shaft, see Supplementary Table S1), a result in agreement with previous estimates^24^. Combining this number with the estimates of age at death and sex for the individualized skeletal remains, this includes at least two children, one adolescent and 17 adults of whom ten are young adults and one middle adult. For the remaining six individuals, the poor preservation allowed only their attribution to an “adult” class (> 20 years old). With one exception (COR-12—extremely fragmentary) we managed to estimate the anthropological sex of all individualized adult skeletal remains, which appear to be mainly males (15/17: 88%).

**Figure 2:**
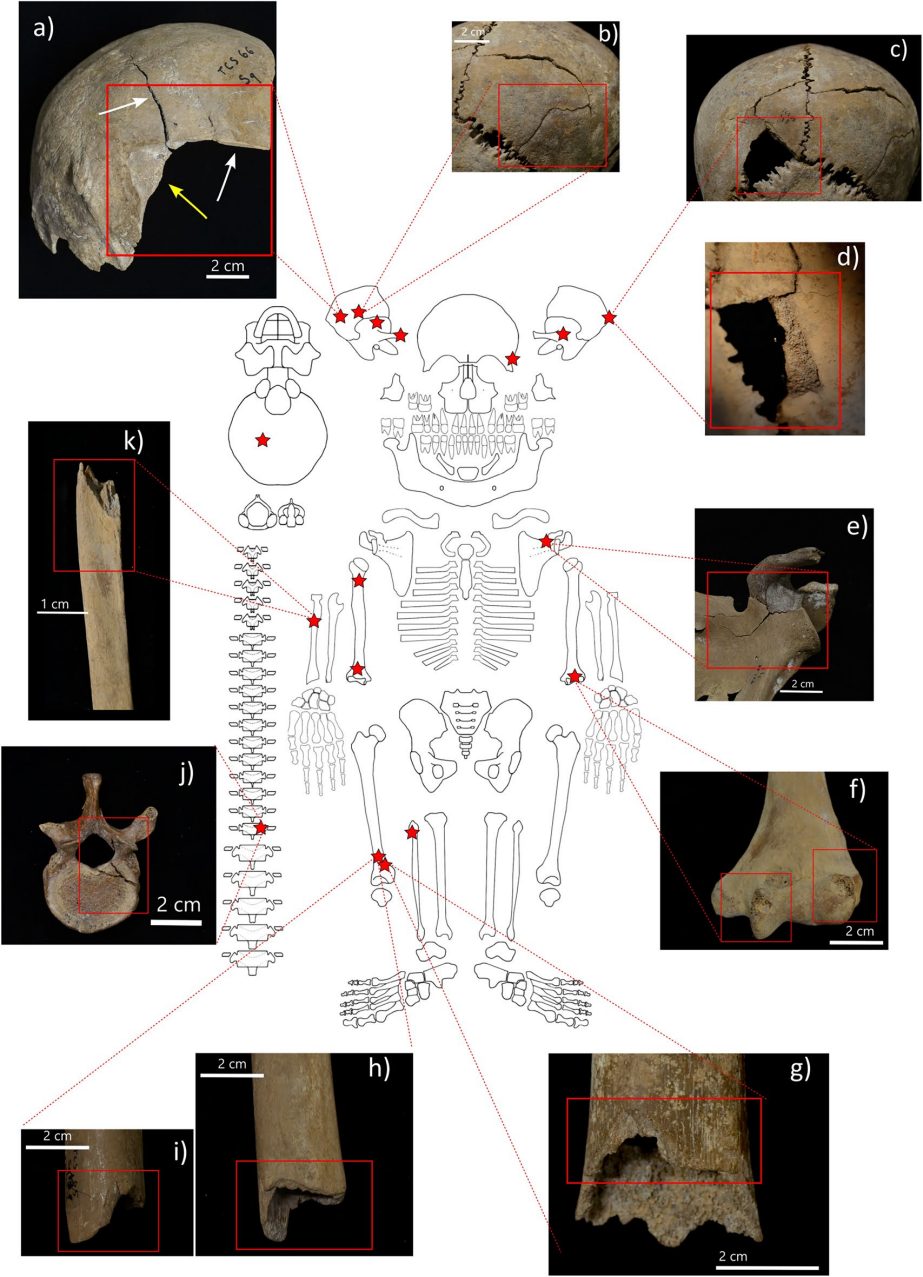
Skeletal distribution and examples of perimortem skeletal lesions at Cornaux/Les Sauges. (**a**) COR-20: blunt force trauma on right parietal and occipital bones associated to two radiating fractures (white arrows) and detachment of ectocranial portion (yellow arrow). (**b**) COR-3: fracture with infero-superior direction on right parietal bone; (**c**–**d**) COR-3: penetrating lesion on left parietal bone (**c**) and endocranial view of the lesion area with beveling (**d**). (**e**) COR-6: left scapula with comminuted impact fracture on the lateral side of left scapula. (**f**) COR-15/17: impacted fractures on the trochlea and capitulum of left humerus. (**g**) COR-12: transverse fracture on distal third of right femoral diaphysis. (**h**–**i**) COR-13: posterior (**h**) and anterior (**i**) view of transverse fracture on distal third of right femoral diaphysis. (**j**) COR-7: crushing fracture on left arc and body of thoracic vertebra (10th or 11th). (**k**) TCS 1489 1972-24-5: transverse fracture on the mid of the diaphysis of an isolated right radius. Skeleton diagram from Roksandic^32^ modified by MM.

A detailed description of the results regarding the paleopathological analyses of the traumatic lesions is provided in Table 1. Ten individuals show at least one perimortem lesion, for a total of 20 observed injuries (Fig. 2, Table 1, Supplementary Table S2). In four cases the same skeleton presents more than one injury on the same or different bones. The lesions are more frequently located on the right side (12/20) and include diaphyseal fractures, impacted fractures, at least one penetrating lesion, and one crushing vertebral fracture. The cranium is the most affected skeletal region (nine lesions), followed by the humerus (five), femur (two), thoracic vertebrae, fibula, scapula, and radius (one lesion each). The morphology of the lesions suggests powerful impact by blunt objects in all cases. One incomplete cranium (COR-20) shows two evident radiating fractures lines (max length of 50 mm) on the right parietal and occipital bones, accompanied by a detachment of part of the ectocranial surface (Fig. 2a).

**Table 1:** Position, morphology, and interpretation of perimortem skeletal lesions.

ID	Bone/Region	N lesions	Description	Force trauma
Sq. 2 CORN 1482	Cranium: right zygomatic arch	1	Detachment and fragmentation	Blunt
Sq. 3 TCS 1483	Cranium: left parietal bone (near lambdoid suture)	1	Penetrating lesion (3 × 2 cm) with endocranial beveling	Blunt
Sq. 3 TCS 1483	Cranium : right parietal bone	1	Fracture with infero-superior direction (length: 5 cm)	Blunt
Sq. 6 TCS 1486	Left scapula: lateral side	1	Comminuted impact fracture on the lateral side of the bone. A single impact caused multiple fracture lines at the level of the glenoid fossa, coracoid process, and scapular blade	Blunt
Sq. 7 TCS 1487	Cranium: right temporal bone	1	Blunt trauma on squamous portion of the bone	Blunt
Sq. 7 TCS 1487	10th/11th thoracic vertebrae (left side)	1	Crushing fracture at left arc and adhering body, fracture goes over left lamina to spinous process	Blunt
Sq. 8 TCS 1488	Cranium: left temporal bone	2	Superior half of the bone (max width: 7 cm)	Blunt
Sq. 8 TCS 1488	Right humerus: surgical neck	1	Surgical neck: transverse fracture on anterior side of the bone	Blunt
Sq. 12 CORN 1492	Right femur: distal third of diaphysis	1	Transverse fracture	Blunt
Sq. 13 CORN 1493	Right femur: distal third of diaphysis	1	Transverse fracture	Blunt
Sq.15–17 CORN 1495	Cranium: left sphenoid and frontal bone	1	Impacted fracture with detachment of bone (area: 3 × 2 cm)	Blunt
Sq.15–17 CORN 1495	Left humerus: trochlea and capitulum	2	Impacted fractures (trochlea: 2 × 1 cm; Capitulum: 1 × 1 cm)	Blunt
Sq. 20 CORN 1500	Cranium: right parietal and right side occipital bone	1	Two radiating fracture lines (rfl) originate from the point of impact, with: 1. antero (mostly)-posterior direction and straight trajectory involving both parietal and occipital bones (max. length 5 cm); 2. super-inferior direction and irregular trajectory (max. length 6 cm). Roughly at the intersection between the rfl there is a detachment of ectocranial bone layer (2 × 2 cm). No endocranial beveling and remodeling is observed	Blunt
Sq. 74 CORN 343*	Right humerus: surgical neck	1	Transverse fracture	Blunt
TCS 1484 Sq.? 1972-23*	Cranium: occipital bone (right side)	1	Detachment of ectocranial surface possible secondary from impact with blunt object (max width: 3 cm)	Blunt
CORN 1498, 1978-138; TCS65-119 G15*	Right humerus: capitulum	1	Impacted fracture (1.5 × 1 cm)	Blunt
TCS 1489 1972-24-15*	Right fibula: proximal third of diaphysis	1	Oblique fracture	Blunt
TCS 1489 1972-24-5*	Right radius: mid- diaphysis	1	Transverse fracture	Blunt

See also Fig. 2 *isolated bones.

Three individuals (bones including one humerus and three femurs) and two isolated tarsals (one talus and one calcaneus) show traces of vertebrate scavenging in the form of pits, punctures, scores, and/or furrows (Supplementary Table S2, Supplementary Fig. S1).

### Radiocarbon analysis

**Figure 3:**
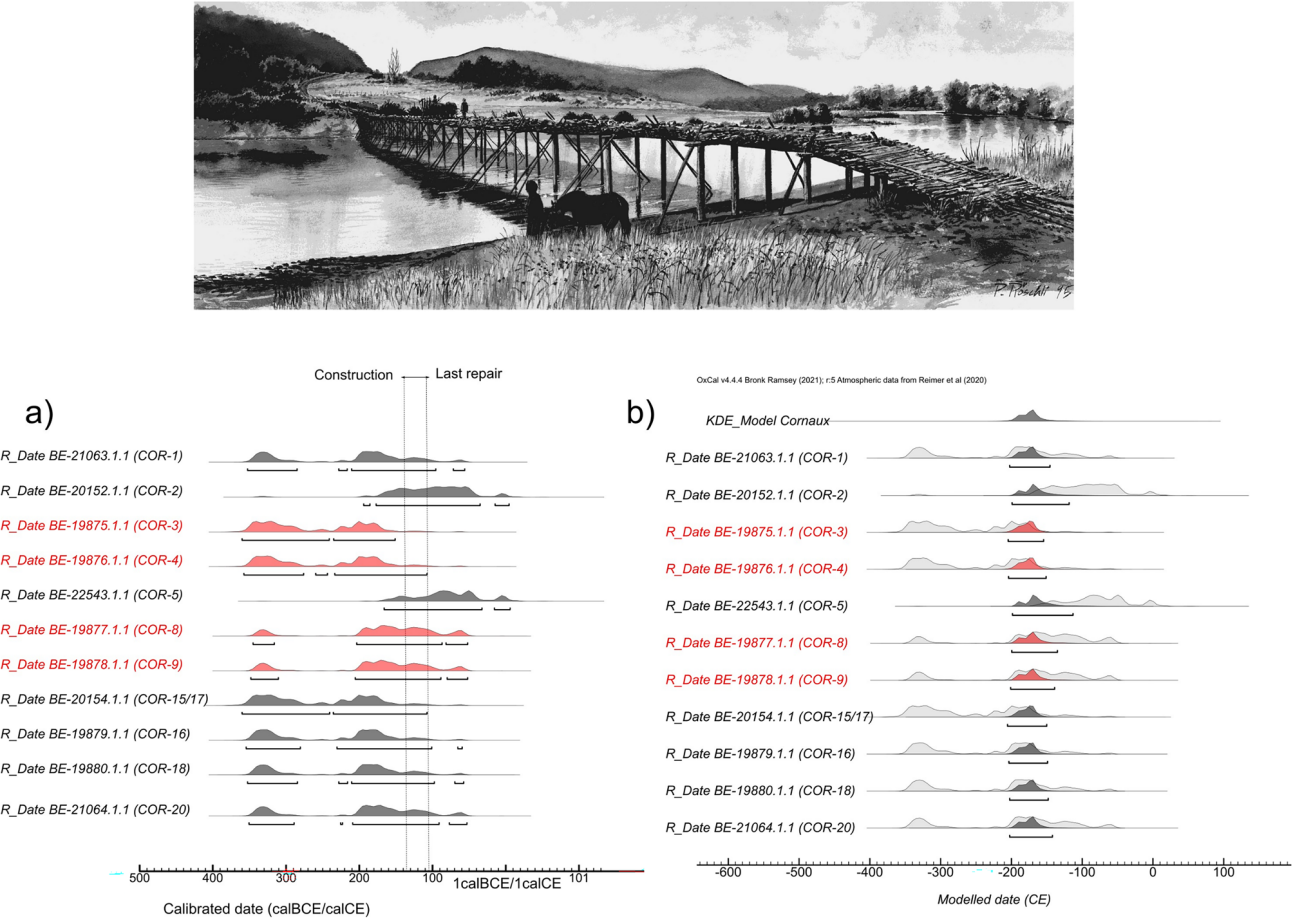
Calibrated radiocarbon data from Cornaux/Les Sauges compared with dendrochronological dates for the construction and last repair of the bridge (dotted lines). Outlier individuals are highlighted in red. Artistic depiction of the Cornaux/Les Sauges bridge by P. Roeschli, courtesy of Laténium—Archaeology Park and Museum Neuchâtel.

New radiocarbon data, added to the dendrochronological estimates for the wood beams, may help better define the processes leading to the observed archaeological assemblage. Eleven out of twelve bone samples fit the quality criteria and provide estimates falling between the 3rd and 1st c. BCE (Supplementary Table S3). The oldest sample (BE-19875.1.1/COR-3) dates to 361–152 cal. BCE (95.4% probability) and the more recent (BE-22543.1.1/COR-5) to 167 cal. BCE–7 cal. CE (95.4% probability) (Fig. 3a).

The model defined by the dendrochronological terminus post quem (TPQ) of 116 and 150 BCE, exhibits poor agreement (A_model = 23.9%) (Supplementary Fig. S2). Four individuals (BE-19880.1.1/COR-18, BE-21063.1.1/COR-1, BE-19876.1.1/COR-4, and BE-19879.1.1/COR-16) display radiocarbon dates that appear much older. Similarly, the model, incorporating all dates with a TPQ of 116 BCE, results in a poor agreement (A_model = 9.3%), with six out of eleven dates appearing too old. Adjusting the TPQ by adding 30 years to account for the average age-at-death of individuals (see methods section) partially improves the first model (A_model = 39.6%).

The model constrained by the chronological extremes of La Tène C2-D1 demonstrates good agreement (A_model = 95.9%), with only two dates (BE-20152.1.1/COR-2 and BE-22543.1.1/COR-5) appearing too recent (Supplementary Fig. S3). Performing a Kernel Density Estimation (KDE) model including all radiocarbon dates and no TPQ also yields acceptable agreement (93.7%), with dates falling between 208 and 117 cal. BCE (median of the KDE model: 175 cal. BCE) (Fig. 3b).

### Isotope analyses

By providing further information about the origin and life style of these individuals, isotopic data on mobility (δ^34^S, ^87^Sr/^86^Sr, δ^18^O) and diet (δ^13^C, δ^15^N, δ^34^S) can offer an additional perspective to assess the various explanations proposed for the site. As shown in Supplementary Table S4, almost all bone samples (38/41; 92.7%) fit the quality criteria for collagen preservation^33^, with the exception of COR-11, AUV-EL1 and AUV-EL3 (bad quality for sulphur), and AUV-EL4 (no collagen yield). All ^87^Sr/^86^Sr and δ^18^O values are reported in Supplementary Tables S5 and S6. Table 2 shows the summary statistics of all isotopic variables considered in this study for human, faunal, plant, land snail shell and water samples.

**Table 2:** Human, faunal, plants and water summary statistics for δ^13^C, δ^15^N, δ^34^S, ^87^Sr/^86^Sr and δ^18^O.

	δ^13^C‰					δ^15^N‰					δ^34^S‰					^87^Sr/^86^Sr					δ^18^Oc‰				
	N	Mean	SD	Min	Max	N	Mean	SD	Min	Max	N	Mean	SD	Min	Max	N	Mean	SD	Min	Max	N	Mean	SD	Min	Max
Humans	19	− 18.9	0.9	− 20.6	− 17.0	19	9.3	1.1	6.9	11.1	18	4.5	2.4	− 1.9	8.0	10	0.71000	0.0016	0.70827	0.71351	10	− 7.9	1.0	− 10.0	− 6.9
*Sus domesticus*	3	− 21.8	0.2	− 22.1	− 21.6	3	5.9	1.2	4.8	7.2	3	3.5	1.4	2.0	4.8	3	0.70871	0.0005	0.70841	0.70932	2	− 9.5	0.4	− 9.8	− 9.2
Herbivores	4	− 21.4	0.4	− 21.7	− 21.0	4	6.2	1.3	4.8	7.8	4	4.2	1.3	3.0	5.6	2	0.70957	0.0012	0.70875	0.71040	2	− 10.2	1.3	− 11.1	− 9.3
*Equus caballus*	2	− 21.7	0.7	− 22.2	− 21.2	2	5.0	0.2	4.9	5.1	2	0.7	0.1	0.6	0.7										
*Esox lucius*	4	− 22.3	0.5	− 23.05	− 21.78	4	11.3	0.3	10.9	11.7	2	2.5	2.3	0.9	4.2										
*Anser anser*	1	− 23.6		− 23.6	− 23.6	1	7.3		7.3	7.3	1	2.9		2.9	2.9										
*Lutra lutra*	1	− 23.2		− 23.2	− 23.2	1	10.8		10.8	10.8	1	2.4		2.4	2.4										
Plants (Rüfenacht)																4	0.70870	0.0007	0.70823	0.70974					
Land snail shells (Rüfenacht)																4	0.70827	0.0001	0.70819	0.70832					
	DIC δ^13^C‰ (PDB)																				δ^18^O‰ (SMOW)				
	N	Mean	SD	Min	Max																N	Mean	SD	Min	Max
Water	3	− 6.3	0.7	− 5.5	− 6.7																3	− 7.7	0.1	− 7.6	− 7.7

*SD* standard deviation, *c* carbonate.

The pig range (mean ± 2SD) for ^87^Sr/^86^Sr is between 0.70766 and 0.70976. These values are consistent with those expected for the Swiss Plateau presented in other paleomobility studies (0.70765–0.70975 in Scheeres^34^; 0.70845–0.71181 in Knipper et al.^35^) and published strontium isoscapes^36^. Furthermore, our values fall inside the isotopic range of the combined plant and terrestrial snail shell samples analyzed in this study from the natural reserve of Rüfenacht, in the neighboring canton of Bern (mean ± 2SD: 0.70745–0.70952; Table 2).

**Figure 4:**
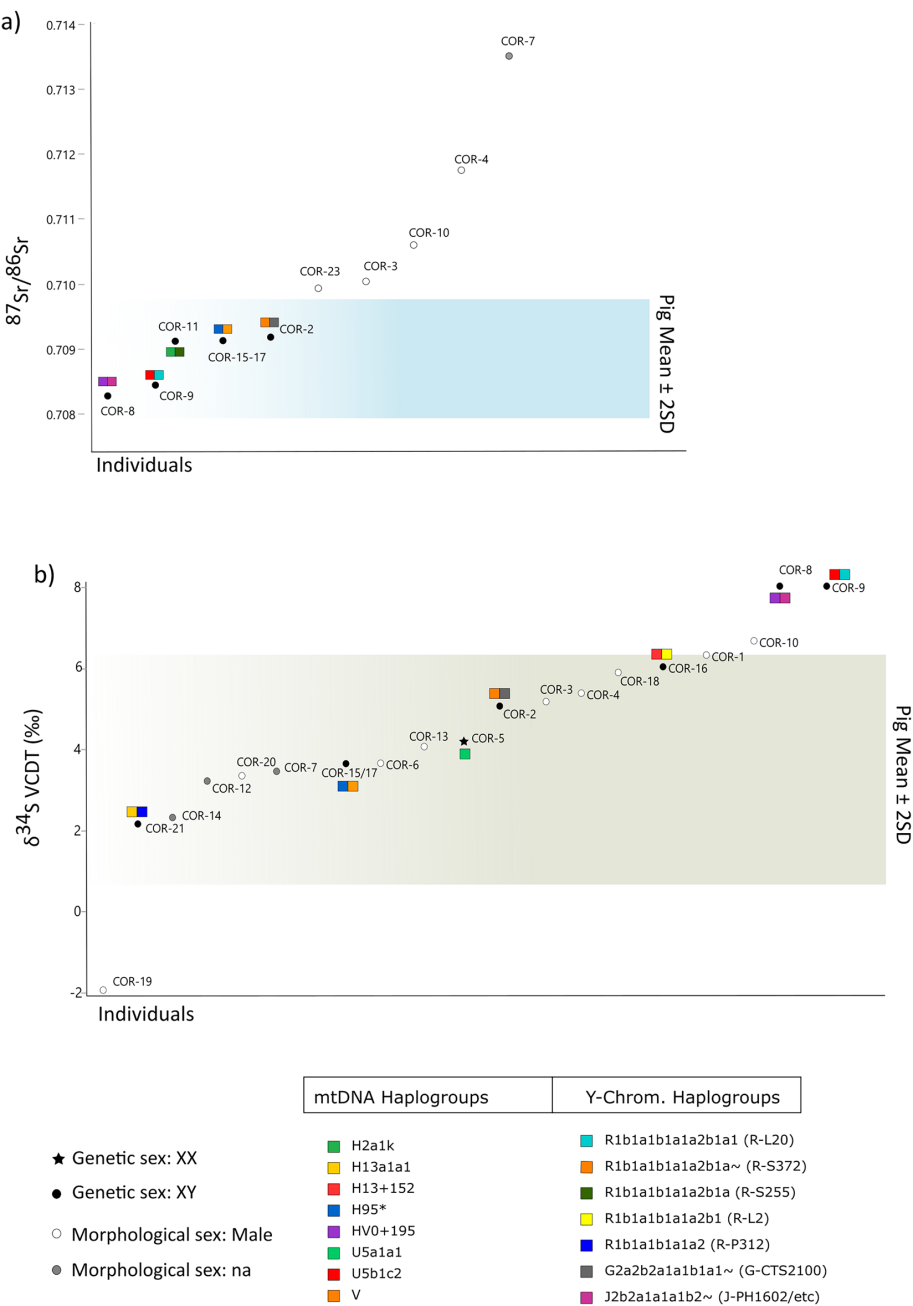
Human isotopic values for strontium (**a**) and sulfur (**b**) compared with the estimated local ranges (shaded areas: pig isotopic means ± 2 SD); integrated with paleogenetic results of the uniparental transmitted markers. Colored rectangles refer to mitochondrial (left half) and Y-Chromosomal (right half) haplogroups. Filled symbols: individuals analyzed genetically. Circles: males; star: female. Empty circles: individuals morphologically male. Grey circles: individuals for whose morphological sex was not assessable.

Measured human values range from 0.70827 to 0.71351 (mean: 0.71000 ± 0.0016). Five individuals present more radiogenic values than the local strontium range (Fig. 4a): COR-7, COR-4, COR-10, COR-3, and COR-23. Regarding δ^34^S, the pig mean ± 2SD range is between 0.7‰ and 6.3‰ VCDT (Fig. 4b), whereas human values range from − 1.9‰ to 8.0‰ VCDT (4.5 ± 2.4‰VCDT). Three individuals (COR-9, COR-8, COR-10) show higher values than the local range, and one (COR-19) a lower sulphur ratio.

δ^18^O isotopic ratios of both available local water samples from the river Thielle are − 7.7‰, while the sample from the Lake Neuchâtel is − 7.6‰. OIPC monthly rainfall estimates for the area of Cornaux range between − 13.1‰ (January) and − 4.0‰ (July).

**Figure 5:**
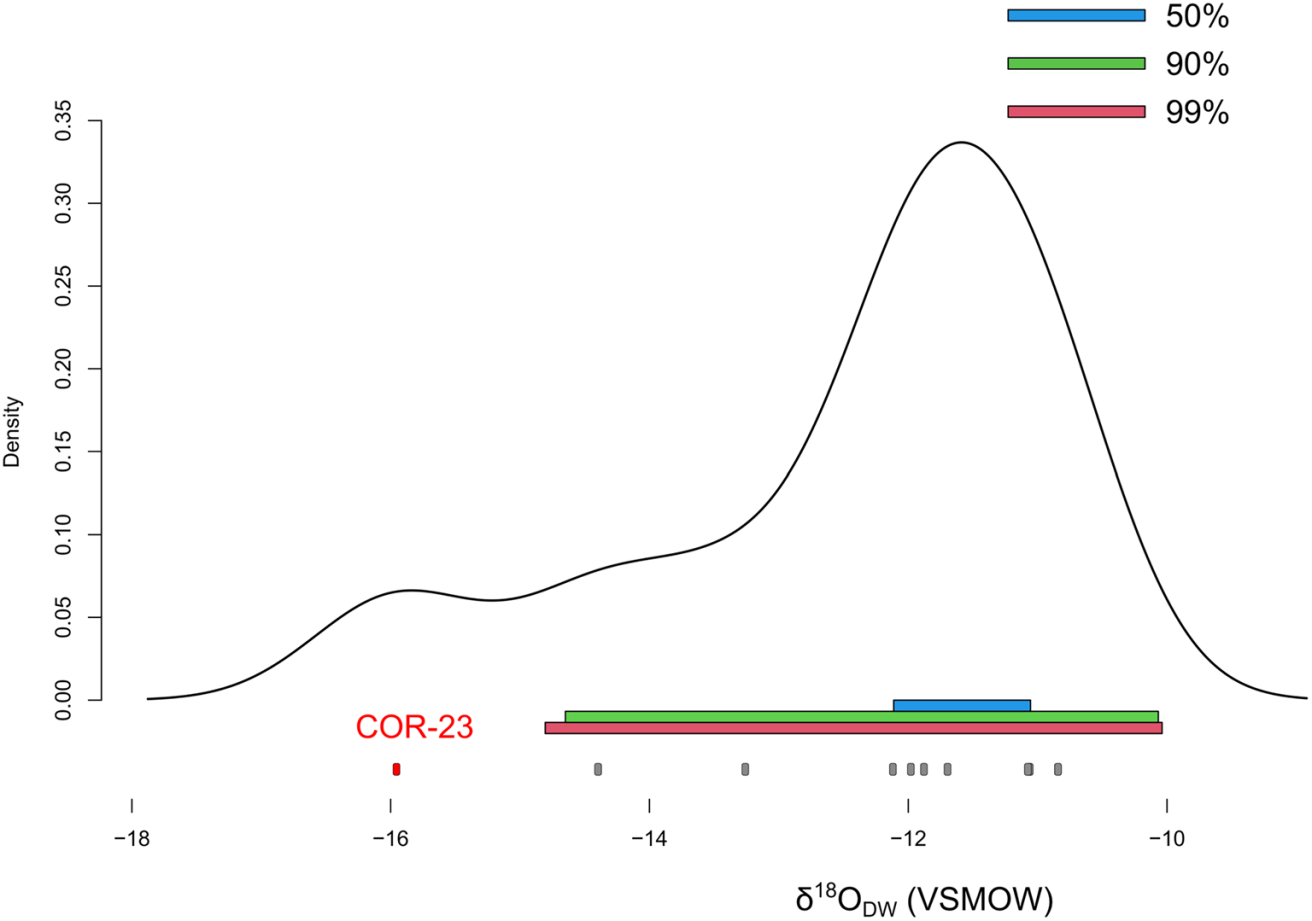
Kernel density distribution of human δ^18^O_DW_ values. Highlighted in different colors are the 50%, 90%, and 99% density regions. The outlier COR-23 is highlighted in red.

After conversion to drinking water (δ^18^O_DW_), the density plot of human oxygen values highlights one outlier (COR-23), characterized by a pronounced lower isotopic value (-15.96‰ VSMOW) (Fig. 5). This individual also falls outside the modern ranges described by the OIPC estimates (Supplementary Fig. S4). The DIC (dissolved inorganic carbon) δ^13^C data for the same water samples fall between − 5.5‰ and − 6.7‰ (PDB).

The offsets between humans and herbivores for carbon, nitrogen, and sulfur is 2.5‰ (δ^13^C), 3.1‰ (δ^15^N), and 0.4‰ (δ^34^S) respectively. Offsets between humans and pigs are 3.0‰ (δ^13^C), 3.4‰ (δ^15^N), and 1.0‰ (δ^34^S). Comparing human and pike the offsets are 3.4 (δ^13^C), -1.9 (δ^15^N), and 2.0 (δ^34^S). The correlation between human δ^13^C and δ^15^N values is positive and statistically significant (Spearman rho = 0.7, *p* = 0.002). When plotted along published isotopic ranges of other protohistoric Swiss contexts (Supplementary Fig. S5a), Cornaux falls within the isotopic variability of other Late Iron Age sites for δ^13^C, although averaging higher δ^15^N values than both Swiss Alpine and Swiss Plateau contexts. No individual shows a pronounced deviation in either carbon or nitrogen values from the others. Apart from COR-3 and COR-10, which show the highest carbon values, the other isotopic outliers for sulfur, strontium, and oxygen do not exhibit specific patterning in carbon and nitrogen ratios (Supplementary Fig. S5b).

### Paleogenetic analysis

Data on genetic sex and relatedness, and population affinity provide additional details about the biological profile of the individuals from Cornaux and the presence of familial relationships among them. This, in turn, offer an additional perspective for discussing this site.

Eleven individuals (11/20, 55%) were suitable for genetic investigation (see Supplementary Text). After the construction of double-stranded genomic libraries using DNA extracts from the petrous part of the temporal bone (PP) (N = 9) and inner ear ossicles (N = 2), samples were tested for their content of endogenous human DNA (human reads—HR) by shotgun sequencing (Supplementary Table S7). Bioinformatics analyses showed that all samples have the characteristic deamination pattern at the end of the DNA fragments (Fig. S6) and low fragment length (average of 72.3 base pairs), indicative of authentic aDNA^37,38^ (Supplementary Table S8). Additionally, they all reached the threshold (≥ 1%) of HR needed for targeted capture analysis and were further enriched for more than 1.3 million single-nucleotide polymorphisms (SNPs) in the human genome and the whole mitochondrial DNA (mtDNA)^39^.

After merging the shotgun and capture sequencing data, the average genome coverage in our samples ranged from 0.1193 to 0.9218 X, while 939,972 to 1,346,808 of targeted SNPs were covered, with a mean coverage spanning from 1.84 X to 10.46 X (Supplementary Table S9). Additionally, most samples (> 81%) show low contamination estimates from modern human DNA (≤ 5% on mtDNA for all samples and ≤ 3% on Y-Chromosome in males), except for two (COR-3 and COR-19; 6% and 24% of contamination on mtDNA level, respectively). After filtering the reads by PMDtools^40^, the two contaminated samples still retained enough human reads for sex determination (810,776 and 1,004,715, respectively)^41,42^ but not for further analyses (kinship, PCA, and clustering analysis). Moreover, the biological kinship analyses (Supplementary Fig. S7) revealed that two individuals exhibited genetic identity (COR-7—left temporal bone and COR-8—right temporal bone) indicating that the bones analyzed from these samples probably belonged to the same individual. Based on morphological inspection COR-7 was excluded from downstream analyses (Supplementary Text).

Therefore, a final dataset of ten individuals (Table 3) was used for sex determination resulting in nine males (XY) and one female (XX), the latter corresponding to a non-adult individual (COR-5). The genetic approach confirmed the sex estimates obtained by osteological inspections in eight adult individuals; however, for two (COR-5 and COR-16), the genetic sex assignment provided new information on their biological sex. Kinship analyses, which were possible for eight samples (excluding COR-3, COR-19, and COR-7) performed using three different methods, consistently revealed a lack of close biological relationships (up to the 3^rd^ degree) among the Cornaux samples (Supplementary Table S10).

**Table 3:** Paleogenetic results of the individuals from Cornaux.

Individual	Sex—Morphological	Age at death	Sex—Genetic	HR—merged data (%)	Mean Coverage—hg19 (X)	mtDNA Haplogroup	Y-Chromosomal Haplogroup
COR-15/17	M	YA	XY	38.68	0.2982	H95*	R1b1a1b1a1a2b1a ~ (R-S372)
*COR-3*	*M*	*YA*	*XY*	*18.64*	*0.0217*	*–*	*–*
COR-11	M	MA	XY	37.49	0.248	H2a1k	R1b1a1b1a1a2b1a (R-S255)
COR-8	M	YA	XY	56.85	0.6972	HV0 + 195	J2b2a1a1a1b2 ~ (J-PH1602/etc.)
COR-9	M	YA	XY	55.4	0.6099	U5b1c2	R1b1a1b1a1a2b1a1 (R-L20)
COR-5	na	C	XX	38.97	0.35	U5a1a1	–
COR-2	M	YA	XY	31.62	0.2064	V	G2a2b2a1a1b1a1 ~ (G-CTS2100)
*COR-19*	*M*	*MA*	*XY*	*16.01*	*0.0228*	*–*	*–*
COR-21	M	Adult	XY	30.06	0.2198	H13a1a1	R1b1a1b1a1a2 (R-P312)
COR-16	F?	MA	XY	51.4	0.7328	H3 + 152	R1b1a1b1a1a2b1 (R-L2)

Individuals included only in the genetic sex determination are reported in italics.

*HR* human reads—human endogenous content, *YA* young adult, *MA* middle adult, *C* child; *na* not assessable, *M* male, *F* female.

These results based on autosomal data were reinforced by those from the unilinear transmitted markers (mtDNA and Y-Chromosome) (Fig. 4). In fact, all samples carried different lineages of the main mtDNA haplogroups (H*, HV*, U5*, and V*) (Supplementary Table S11). Comparative analyses reveal that most lineages have been found in individuals from England but also Czech Republic and Italy^43,44^. One lineage (H3 + 152) has been found only in Iron Age Spain^45^ and one haplogroup (H95* in COR-15/17) has never been described before has never been described before according to the Allen Ancient DNA Resource (AADR)^46^ dataset (compared to 10,066 ancient individuals from the dataset). Similarly, paternal Y-Chromosomal haplogroup assignment found three different macro haplogroups (R1b*, G2a*, and J*) with a prevalence of R1b* (71%), which includes different lineages (e.g., R-S372, R-S255, R-L20, R-S27458, and R-L2) (Supplementary Table S11) confirming no close relatedness at a paternal level among the males analyzed. However, missing data or low-quality DNA of the Y-Chromosome should be considered when evaluating these results. The absence of the mutation R-L20 at the Y-Chromosome position [14231292] in COR-11 (R1b1a1b1a1a2b1a (R-S255)) may have prevented the assignment of the more derived R-sub lineage R1b1a1b1a1a2b1a1 (R-L20) of COR-9, and therefore a match cannot be excluded.

**Figure 6:**
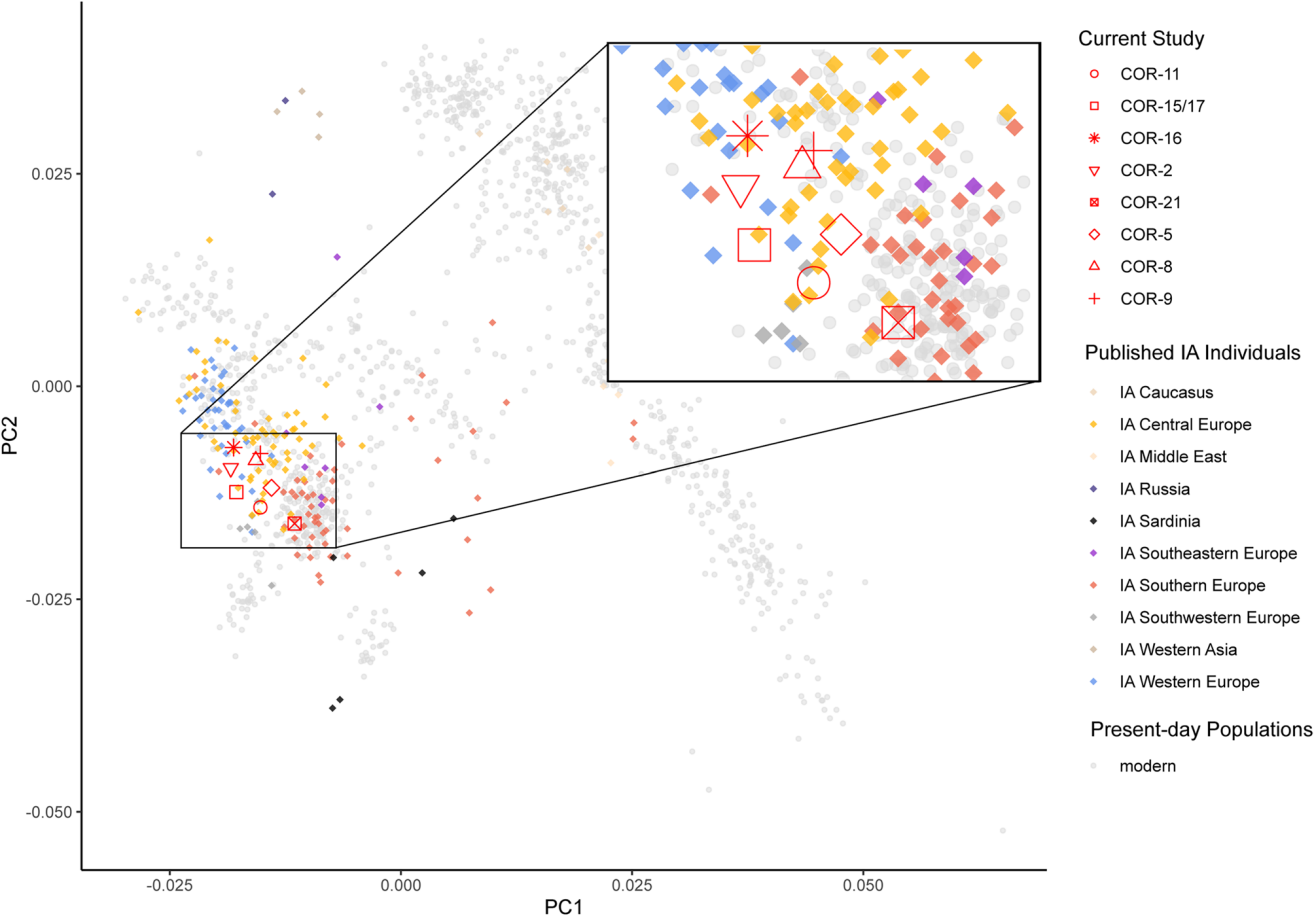
Principal component analysis of ancient samples from Cornaux and other published ancient individuals from the Iron Age (IA) projected onto the genetic complexity of present-day individuals of western Eurasia. Individuals from Cornaux are represented in red symbols whereas circles are used for present-day individuals and diamond-shaped symbols represent published ancient IA individuals from Europe. Different colors indicate the geographic origin.

Comparative analyses show that to get a first picture of the genetic relationships of our samples and other ancient individuals, we performed a Principal Component Analysis (PCA). When the ancient genomes from Cornaux are plotted alongside published data of other Iron Age individuals (“IA”—8^th^—1st c. BCE) from western Eurasia on a PCA of data from present-day European populations (Supplementary Table S12), the plot shows that most Cornaux individuals show a genomic affinity with each other and with IA samples from Western (France^43^) and Central Europe (e.g., Hungary, Czech Republic, Slovenia, and Slovakia^43,47^). Only one (COR-21) differs slightly from the others by overlapping on the plot with IA individuals from Southern Europe (Central Italy^44,48^) (Fig. 6 and Supplementary Fig. S8; Supplementary Table S13). Accordingly, unsupervised cluster analysis (Supplementary Fig. S9) reveals that the Cornaux samples exhibit similar genomic structure and ancestry components when analyzing the three main ancestral components that contributed to the genetic makeup of present-day Europeans (Hunter-Gatherer-related ancestry, Neolithic farmers-related ancestry, and, finally, Steppe-herders-related ancestry^49,50^).

Supplementary Table S14 presents an overview of the demographic structure, isotopic, genetic, depositional, and taphonomic data for the individualized skeletons.

## Discussion

The aim of this work was to evaluate the two explanations typically proposed for Cornaux/Les Sauges (accidental event vs. executions/sacrifices) based on new osteological, isotopic, and ancient DNA analyses of the human remains from this site.

The primary challenge when comparing our anthropological and radiocarbon data with the two proposed scenarios for the site is the lack of distinct features that allow favoring one hypothesis over the other. At the same time, when considered together, the diverse data from this study offer new insights into the most plausible explanation.

An accidental event involving a random group of people would theoretically lead to a heterogeneous representation of sexes and age classes^51^. Thus, the fact that the majority of the individuals from Cornaux are young or middle adult males would militate against this scenario. The recovered skeletons would rather represent a situation where a sex bias can be expected. Possibilities include sacrifices or executions of slaves, defeated warriors or war captives, or an accidental event involving a convoy of warriors or traders passing on the bridge at the time of the collapse.

Both the interpretive models proposed for the site imply a chronological proximity between the recovered skeletons and the construction of the bridge. The unexpectedly older ^14^C dates of some individuals may stem from various factors. First, they might reflect different events through time, e.g., accidents and/or executions, or a combination of events. Another explanation is that of a combined effect of different variables. First, the wiggling of the calibration curve between 350 and 200 BCE, which adds a larger error for the time period under study. Second, ^14^C data from bone collagen reflect the life course, not the postmortem interval. Paired with the possible time gaps between felling dates and the construction/ repairing of a structure, shifts between these estimates are expected. Finally, we need to consider the possibility of a freshwater reservoir effect (FRE)^52–54^. The latter is known to potentially lead to dates appearing a few hundred to thousands of years older^54,55^. DIC δ^13^C values measured in water samples from Lake Neuchâtel and the Thielle River suggest the presence of ^14^C-free (‘dead’) carbon in these freshwater systems. The latter may originate from the dissolution of carbonates or CO_2_ inputs from carbonated waters^53,56^. This is not completely unexpected as calcareous sediments characterize the geological environment of the northern area of this lake^57^. It should be noted that stable isotope ratios of carbon and nitrogen of Cornaux do not suggest a substantial consumption of freshwater fish by these individuals. On the other hand, this conclusion is based on the comparison of human isotopic values with those from archaeological pike remains from the Neolithic settlement of Auvernier/La Saunerie^58^, and an archaeological otter from Cornaux. Freshwater systems are known to be isotopically quite variable^59–61^. This, combined with the possible nonlocal origin of some of the individuals, raise the possibility that our analyses are unable to trace this dietary component.

The scavenging traces observed on only a few isolated or disarticulated remains suggest that corpses were, in general, only partially accessible for scavengers and that this may have happened only when body parts emerged after decomposition^62,63^. This evidence together with the documented preservation in some cases of brain remains, allows the exclusion of a postmortem exposure of bodies on the bridge, decaying in the open air until advanced decomposition^64,65^. On the other hand, the same finds may in principle be consistent with both a catastrophic event leading to the submerging and “pinning” of the corpses underwater, as well as with the throwing of sacrificial/execution victims tied to weights into the water. This would also explain the absence of abrasions that are expected when remains are subjected to some degree of movement on a river bed^66^. The preservation of brain tissues in five cases, and, in at least seven skeletons, of the anatomical connections would be unexpected assuming a decomposition in aquatic environment^63^. Rather, it would better fit a scenario where bodies are quickly covered by sediment leading to a humid and anaerobic postdepositional environment. Paired with the association between skeletons and bridge elements, these considerations would therefore support an accidental event.

Regarding the observed traumas, clinical data report that skeletal lesions experienced by survivors of a tsunami event (one of the hypotheses postulated for Cornaux^28^) mostly include blunt and crush forces injuries, with a higher frequency of lesions affecting the lower limbs^67,68^. Conversely, victims of sacrifices and executions are more likely to show sharp force lesions in conjunction with blunt force injuries^69–71^. Although variable, the distribution of lesions is expected to cluster at the level of the typical target of interpersonal violence, e.g., the cranium, mandible and, especially in cases of executions, the upper cervical vertebrae^72–74^. All in all, the investigated traumas at Cornaux do not necessarily rule out the performance of sacrifices or other violent activities perpetuated through blunt weapons^74^. However, the absence of any clear intentional lesion, the heterogeneous anatomical distribution of the injuries and the substantial force suggested by the latter fit overall an accidental scenario rather than one characterized by intentional violence. Collapsing bridge elements, therefore, may have hit and crushed some of these individuals.

Another potential problem affecting a univocal accidental explanation is the relatively high frequency of lesions on crania and mandibles, which deviates from observations on victims of catastrophic (tsunami) events. Kaewlai et al.^67^ postulate however that the underrepresentation of some types of lesions, such as cranial fractures, could be attributed to their infrequent occurrence among survivors who seek medical attention at hospitals. Including data from individuals who did not survive may alter the reported frequencies.

Further support for the hypothesis of an accident is provided by the faunal remains (from cattle and horses) found in partial anatomical connection on the lower bank of the river and on the riverbed in association with the human articulated skeletons, and generally lacking those lesions expected in the case of sacrifices and/or butchering. The only sharp force injuries (two, on a horse rib) in this case have been interpreted by Mèniel as accidental^cf.^^12^. Remains from other domestic animals, e.g., pigs and caprinae*,* were retrieved in the upper part of the riverbank and in the area defined as “habitat” by Schwab^13^ and are mostly represented by scattered and fragmentary bones showing butchering marks in some cases^75^. The zooarchaeological and anthropological data, therefore, would converge toward the same type of explanation.

Both the hypotheses of a convoy involved in a natural catastrophic event and that of sacrifices/executions of war captives and/or slaves imply the possibility of some of the victims being nonlocals. The combination of strontium, oxygen, and sulfur data results in a range of outliers between five and eight out of 20 (25–40% of the sample). These frequencies are not dissimilar to those observed in culturally and chronologically similar contexts, and may reflect a generalized pattern of high territorial mobility during the European Late Iron Age^34,35,76–81^. While isotopic data are informative about one individual’s mobility during life, the variability of the mtDNA haplogroups indicates, on a deeper chronological scale, a possible different genetic origin of the analyzed individuals. The results of the PCA point to a close genetic affinity of almost all our analyzed samples to Iron Age groups from Western and Central Europe, as expected independently from the events taking place at the site.

We may anticipate that a group of people being on a long-term expedition would be accompanied by some family members. The hypothesis of a convoy running into a natural disaster, therefore, would imply the likelihood of detecting a genetic relatedness among at least some of the analyzed skeletons. The results of our analyses, however, do not align with this expectation thus countering against the hypothesis of a passing convoy. The absence of any close genetic kinship, paired with the sex bias of the individuals, can therefore not exclude the possibility of a specific group, including their identity as potential victims of execution or sacrifices. However, we need to consider that, first, we have data on genetic relatedness only for eight out of 20 of the skeletons. Second, the recovered skeletons are likely a subsample of the original number of individuals. The latter would have included the possible survivors as well as casualties whose skeletal remains were dispersed and not recovered.

The obtained isotopic and paleogenetic data hold broader relevance for the discussions of mobility, diet, and genetic variability in Late Iron Age Switzerland. Isotopic data on mobility and diet are consistent with previous results from other Late Iron Age contexts, suggesting a substantial degree of mobility. Regarding the geographic origin of the nonlocal individuals, we may postulate some exploratory hypotheses based on their strontium and oxygen values. The more radiogenic signal of the outliers for strontium, especially COR-4 and COR-7, is consistent with a childhood spent in areas featuring older metamorphic and magmatic rocks. Limiting ourselves to a range of ca. 100–150 km from Cornaux, this may include the Alpine range, as well as Southern Germany. Intriguingly, all these regions correspond to the core of the geographic extension of the La Tène culture^82^. They would therefore fit a scenario of intense demic and cultural networks among different “Celtic'” groups in this period, as also suggested by the archaeological record^77^. However, caution is required when discussing the possible origin of the nonlocal individuals, as this is a task that would require a larger and more spatially dense dataset. It is nevertheless interesting the fact that a possible childhood spent in an Alpine region would also explain the low δ^18^O value of COR-23. We need however to be aware of the possibility that this lower ratio may be due to the ingestion of water originating from, and preserving the isotopic value of a colder, higher altitude area^83^.

Isotopic data from Cornaux also fit previous estimates highlighting the dietary relevance of both C_3_ (e.g., wheat, barley) and C_4_ (e.g., millet) plants in this period and area, while suggesting a variable exploitation of animal proteins^84–86^. Conversely, and despite the positive correlation between δ^13^C and δ^15^N values, the consumption of freshwater fish seems to have been negligible. This is suggested by the low δ^13^C from the archaeological pikes and the reduced offset between humans and pike for δ^15^N (− 1.9‰) as well as the isotopic values of the otter, whose diet mostly includes fishes. Following this reasoning, the relatively high carbon values shown by COR-3 and COR-10 would therefore stem from of a greater consumption of C_4_ plant products. This in turn is interesting given the possible alpine and subalpine origin earlier suggested for these individuals. Previous analyses have indeed indicated a relatively higher exploitation of millet in Alpine areas in Switzerland during the Final Bronze Age^87^ and Late Iron Age^86,88^, a custom also shared by “Celtic” groups from subalpine areas of North-eastern Italy^89^. However, we need to be cautious in excluding consumption of freshwater fish since the fish remains used for comparison here come from a different chronology (Neolithic) and place (Lake Neuchâtel) than the river Thielle, and considering the mentioned isotopic variability of freshwater trophic systems.

An important aspect of this study is that it offers for the first time a glimpse into the genetic structure and variability of Late Iron Age Switzerland. The high variety of mtDNA haplogroups of the samples of Cornaux indicates a high genetic variability within these individuals. Additionally, the found mtDNA haplogroups are all observed in other European Iron Age samples^43,44,47,48^ except for one (haplogroup H95*; COR-15/17) which has never been described up to date. Moreover, comparative analyses highlight a close genomic affinity of most of our analyzed samples to Iron Age groups from Western and Central Europe with only one individual (COR-21) showing genetic affinity to Iron Age samples from Southern Europe (Italy), suggesting a possible different genetic history for this adult male.

The paleogenetic data from Cornaux are therefore in agreement with other ancient DNA studies on samples from the same period^43,90^ which suggest genomic affinity among Iron Age groups from Europe. Additional data and analyses (in progress) will provide the opportunity to better understand the genetic makeup of Late Iron Age Switzerland.

## Methods

### Osteological analysis

#### Minimum number of individuals (MNI)

The MNI was estimated applying protocols described in White^91^ and in Buikstra and Ubelaker^92^. We first subdivided all available skeletal elements—independently from their attribution to individualized skeletons—according to their side and separated adult from non-adults remains. We then counted each element, and, in the case of fragmentation, noted which part of the bone was preserved (e.g., proximal end, proximal, middle or distal third of the shaft, distal end) and coded the degree of completeness (see Supplementary Table S1). In order to avoid possible bias on the estimate of misidentified sides or order, this step did not include vertebrae and ribs except for the first two cervical vertebrae and the first rib on either side, and phalanges. We then used the highest number of the most repeating element as the MNI.

#### Estimation of age at death and sex

Adult age-at-death was estimated according to the standard methods collected in Buikstra and Ubelaker^92^, i.e., morphological changes of the pubic symphysis and auricular surface of the ilium, and, in case of absence of the pelvic bones, we observed the degenerative changes of the sternal end of the left 4th rib or other ribs. Non-adults’ age-at-death was estimated based on the development and eruption of deciduous and permanent teeth^93^ and on the degrees of epiphyseal fusion^94^. We indicated the age at death of the individuals following the categories proposed in Buikstra and Ubelaker^92^: fetal-F (< birth); Infants-I (birth-3 years); children-C (3–12 years); adolescents-Ao (12–20 years); Young adults-YA (20–35 years); Middle adults-MA (35–50 years); Old adults-OA (> 50 years).

Sex was estimated based on the morphology of the pubic symphysis, coxal bones, and cranial and mandibular dimorphic traits, following standard anthropological methods^92^.

#### Skeletal traumas and taphonomic analyses

We screened all available bones, including scattered remains, for the presence of traumatic lesions macroscopically with the aid of a magnifying glass and microscopically using a Dinolite USB digital microscope. We recorded traumas according to their timing, type, length, width, and anatomical location. Metric measurements were taken using a sliding caliper. The timing of the traumatic event separates intravitam (healed, during lifetime) from perimortem (around the time of death) and postmortem lesions (after death, during an advanced state of skeletonization). Features defining perimortem traumas include the lack of traces of bone reactive processes, a shape and edges suggestive of impact on a relative plastic skeletal element, and a coloration of the lesion similar to that of the surrounding bone^95,96^. Postmortem traumas differ from perimortem based on their morphological features consistent with a biomechanical strain to a rigid, dry bone tissue and their coloration, usually lighter than that of the surrounding bone. Following standard approaches from forensic anthropology, we then classified the observed lesions according to their origin in blunt force, sharp force, or penetrating lesions^97^.

The taphonomic analysis included the visual examination of each bone (N = 959) and documentation of traces left by animal and plant activity (e.g., gnawing marks, root etching), abrasion due to water movement, particular discoloration and modification of the bone cortex due to changes in humidity, and postmortem breakages in wet versus dry bone among others following the suggestions of Binford^98^, Evans^66^, and Pokines and Jans^99^. We then calculated the frequency of perimortem fractures and of scavenging activity by individual (excluding therefore the isolated remains) and by skeletal element.

### Isotopic and radiocarbon analyses

#### Sampling and isotopic baseline estimation

Our sampling strategy aimed at maximizing the comparability between isotopic, genetic, and anthropological data trying to minimize invasiveness (Supplementary Fig. S10). The sampling targeted those remains originally identified during excavation as pertaining to separate (numbered) individuals. However, it is worth stressing that some of these lack the left femur (the bone used for our estimation of the MNI), while having teeth and/or temporal bones. This prompted us to sample cranial bones if the left femur was missing. Conversely, teeth were available only for a few individuals who may have lacked postcranial elements. All these issues led to different sample sizes for the analyses involving bone collagen (N = 20) and dental enamel (N = 10).

Since genetic analyses revealed that samples COR-7 and COR-8 (respectively right and left pars petrosa) pertain to the same individual (see section Paleogenetic Analyses), we excluded the right pars petrosa COR-7 from the carbon, nitrogen, sulfur, and ^14^C analyses, thus reducing this dataset to 19 individuals. The same issue does not affect the analysis of dental enamel since COR-7 and COR-8 have mandibles with teeth, which protects against the risk of double sampling the same individual.

In order to better contextualize the isotopic data from Cornaux, we also collected bone samples from five individuals (four adult males and one adolescent) from the nearby (3 km) site of La Tène (4th–1st c. BCE).

We submitted the collagen obtained from these samples for carbon, nitrogen and sulfur stable isotope analysis. Of these, eleven were also processed for ^14^C analysis. The ten dental enamel samples were analyzed for strontium and oxygen isotopic ratios (see Supplementary Tables S5 and S6 for the composition of each dataset).

To estimate the local range of bio-available δ^13^C, δ^15^N, and δ^34^S, we collected 16 faunal bone samples (three cattle, two horses, three pigs, one caprine (goat/sheep), one goose, one otter, and five pikes). All faunal remains come from the Cornaux site, with the exception of the pikes which came from the Final Neolithic-Bronze Age site of Auvernier/La Saunerie^57^ on the Lake Neuchâtel ca. 13 km away.

Lacking vegetation or soil samples from the analyzed area we estimated the local strontium isotopic range based on three archaeological pig teeth from Cornaux. Pigs, as well as micromammals and terrestrial snails, are often used in provenience studies as a proxy of the local isotopic values due to the fact that they are often kept and fed locally^100,101^. The diet of domestic pigs is indeed similar to that of humans^102^, including typically remnants of human activities like decomposing vegetables, food leftovers and waste from both humans and animals^100^. One possible issue of this approach is that pigs may have been imported from somewhere else, resulting in a biased estimate of the local isotopic range. In our case, we tried to minimize this risk by comparing the isotopic values of these animals with published ranges for the Swiss Plateau^34,35^, considered here as the local range, as well as the analysis of vegetation (n = 4) and terrestrial snail shell (n = 4) samples from Rüfenacht (Bern). The latter is a natural reserve relatively close (ca. 33 km) and geologically similar (Swiss Plateau molasse basin) to Cornaux. Finally, our local range was further compared with available strontium isoscapes for Europe^36,103^ as well as a survey of geological maps for the study area (https://www.swisstopo.admin.ch/).

The suitability of environmental and faunal data for reconstructing local δ^18^O has been criticized^87^ based on a large range of factors possibly biasing the obtained estimates. For this reason, we first checked the distribution of δ^18^O in the human sample by means of a Kernel density estimation (see also^83,104,105^) and used the range of the 95% density region as a threshold for defining outliers (see^105^ and Supplementary text: isotopic analyses). The most obvious limit of this approach is the small sample size (N = 8), an issue that we took into account when discussing our results.

We then discussed the results of this exploratory analysis based on the oxygen values obtained from the analysis of 3 surface water samples collected in November 2022 near Cornaux (river Thielle, n = 2 and the Lake Neuchâtel, n = 1), published δ^18^O precipitation isoscapes for Europe, and estimated monthly and annual precipitation values for the region under study. The latter, obtained from the Online Isotopes in Precipitation Calculator (OIPC), is available at waterisotope.org^106–109^.

#### Bone collagen and enamel carbonate preparation, isotopic analysis

The extraction of collagen was performed in the Department of Physical Anthropology of the University of Bern following an acid–base–acid extraction method modified after Ambrose^110,111^, DeNiro^112^, and Longin^113^. The isotope ratios of carbon (^13^C/^12^C), nitrogen (^15^N/^14^N), and sulfur (^34^S/^32^S) were measured by isotope ratio mass spectrometry at Isolab GmbH, Schweitenkirchen, Germany (see Supplementary Methods: isotopic analyses). The preparation of the enamel samples for oxygen and strontium isotope ratio analyses and plant and snail shell samples for strontium was conducted in both the ANTARQBIO lab of the Universitat de València (Spain) and the Department of Geological Sciences of the University of Cape Town (South Africa). The enamel sampling was preferentially performed for both elements on the buccal side of the tooth after the sampling and the photographic documentation of dental calculus when present. The selected crown surface was cleaned by abrasion with tungsten drill bits and the enamel powder samples for Oxygen analyses were removed by drilling with a diamond bit, along a line down the tooth crown from the occlusal surface to the enamel-root junction. A low-magnification lens (× 3) was used during the whole sampling process in order to avoid collecting any superficial cement on the tooth or dentine underlying the enamel^114^. Samples weighing (4.5–9 mg) were chemically treated following protocols originally proposed by Lee-Thorp and van der Merwe^115^, and modified by Koch et al.^116^, Balasse et al.^117^, and Tornero and colleagues^114^ (see Supplementary methods: isotopic analyses). The measurements of DIC δ^13^C, δ^18^O (and δD) of the collected local water samples were performed in the Stable isotopes biogeochemistry laboratory of the Andalusian Earth Sciences Institute of Granada (Spain) (see Supplementary methods: isotopic analyses).

For the Strontium analyses, we sampled a small chunk of enamel from human and animal teeth using a diamond drill bit with a Dremel 3500 on the same side as the enamel powder sample for oxygen analyses. The portion of sampled enamel (ca. 20 mg) was cleaned by abrasion and possible dentine remains were removed using a tungsten drill bit. Samples were finally rinsed with MilliQ water, ultrasonicated for 15 min and left to dry. A low-magnification lens (× 3) was used during the whole sampling process in order to avoid collecting any superficial cement on the tooth or dentine underlying the enamel. All diamond and tungsten drill bits used during this process were cleaned with ethanol, rinsed and ultrasonicated in MilliQ water during 15 min between samples to avoid cross-contamination^118^. The pre-treatment of plant and snail shell samples is described in the Supplementary text. The chemical treatment and separation chemistry followed methods described in Pin et al.^119^ (see Supplementary methods: isotopic analyses). Radiocarbon dating of collagen samples was carried out at the LARA laboratory at the University of Bern according to Szidat and colleagues^120^. Two samples were repeated in the Tandem Laboratory within the Faculty of Science and Engineering at Uppsala University. For a detailed description of the protocol and equipment, see the Supplementary methods: isotopic analyses. Radiocarbon ages were translated into calendar ages with OxCal 4.4.4^121^ using the IntCal20 calibration curve^122^. In an attempt to better refine the individual radiocarbon dates we ran a KDE model in Oxcal including all radiocarbon dates. This attempt was followed by additional models: (1) considering only those individuals associated to dendrochronologically analyzed wood beams, and performing a model according to two overlapping phases, each one constrained by the dendrochronological dates of the beams (116 and 150 BCE) as terminus post quem (TPQ); (2) a model that includes all individual radiocarbon dates and use a TPQ of 116 BCE (the most recent estimated felling date). Considering that ^14^C does not reflect the exact time of death of an individual, and considering that the majority of skeletons show an age-at-death between 20 and 35 years, we performed the above models twice, the second time adding 30 years to the TPQ. (3) a model for a single phase constrained by estimated chronological extremes of La Tène C2-D1 (based on the typology of the objects recovered from the site, ca. 200–80 BCE).

### Genetic analyses

#### Sample selection and sampling, DNA Extraction and Library Preparation

Eleven individuals were selected for aDNA analysis based on preservation and availability of the petrous part (PP) and ear ossicle bones. PP samples were obtained by drilling the petrous pyramid (approx. 200 mg) while whole middle ear bones (approx. 80 mg) were collected for DNA extraction. After DNA extraction following a silica membrane-based method^123,124^, the extracted DNA was converted into double-stranded, double-indexed Illumina libraries modified from^125^.

#### Molecular screening and in-solution target enrichment

The indexed libraries were shotgun sequenced at the facility Macrogen (Seoul) on a HiseqX platform (150 bp PE). All eleven samples fulfilled the quality criteria (presence of damage pattern of aDNA and content of human endogenous DNA ≥ 1%) and were then enriched for more than 1.3 million human SNPs using the “Twist Ancient DNA”—kit (Twist Bioscience) with a modified protocol optimized for aDNA^39^. Subsequently, the enriched libraries were sequenced again on an Illumina Hiseq X (150 bp PE) system at the Macrogen Sequencing Centre (Seoul).

#### Bioinformatic analyses and aDNA authentication

Bioinformatic analyses and aDNA authentication were conducted using sequences generated via shotgun sequencing of Whole Genome Sequencing (WGS) libraries and sequences generated separately via sequencing of enriched libraries. After trimming and merging^126^ the reads, they were aligned^127^ to the Human Reference Genome (GRCh37/hg19) and the revised Cambridge Reference Sequence (rCRS). We removed duplicates (DeDup, v. 0.12.8^128^), examined damage patterns in the ancient reads (mapDamage, v. 2.2.1^38^), and estimated modern human contamination at both nuclear (only for males—ANGSD^129^) and mitochondrial (for all individuals—Schmutzi^130^) levels with thresholds of ≤ 3% and ≤ 5% respectively. For samples that did not pass the quality criteria, we applied PMD tools^40^ with a quality threshold of 30 to remove reads possibly attributable to the contaminants. For all samples that passed the applied quality criteria, we merged shotgun and target-enrichment data for the performance of downstream analyses. Quality statistics are summarized in Supplementary Tables S8 and S9.

#### Sex determination, unilinear transmitted markers, and kinship analyses

Biological sex was determined on 10 samples by applying two different methods^38,39^. The mtDNA haplogroup was assigned with HaploGrep3 (3.2.1) (https://haplogrep.uibk.ac.at/; based on PhyloTree, mtDNA tree build 17, available via www.phylotree.org/)^131^. We used the consensus file from Schmutzi as the input files, with the exception of two samples (COR-15/17 and COR-21) for which .vcf files were used. The Y-Chromosomal haplogroup assignment in males was inferred by the Yleaf software^132^ (ISOGG; Version: 15.73 Date: 11 July 2020).

Biological relatedness (kinship) among the individuals from Cornaux was inferred by using three different methods developed especially for low-coverage aDNA data: READ, TKGWV2, and KIN. The first two methods (READ, Relationship Estimation from Ancient DNA^133^ and TKGWV2^134^) allow us to infer genetic relatedness only up to the 2nd degree. On the other hand, KIN^135^ can identify kinship up to 3^rd^-degree and distinguishes between sibling and parent–child relationships.

#### Comparative analyses and dataset

Merged data (shotgun + capture) of 8 non-related samples from Cornaux presenting more than 50,000 overlapping SNPs on the 1240 k dataset was used for genome-wide downstream analyses. After genotyping (samtools mpileup^136^) the samples, we reconstructed pseudo-haploid genotypes (pileupCaller; https://github.com/stschiff/sequenceTools) eliminating transition sites to minimize errors caused by postmortem damage.

In order to examine the genetic relationships between the individuals from Cornaux and other present-day and already published ancient individuals from Europe, Principal Component Analysis (PCA) was performed. For this, the genotyped samples from Cornaux were merged with the HO dataset (AADR, version 54.1; November 16th 2022^46^) described in the Supplementary text. We then projected the ancient individuals (N = 473) onto the genetic variability of present-day Eurasians (N = 1575) using the lsqproject option and shrinkmode parameter from smartpca^137^.

We applied unsupervised cluster analysis by using ADMIXTURE^138^ to analyze the genetic structure of the individuals from Cornaux. To do this, we used the same genomic data of the HO_dataset, which was subjected to variant pruning to address linkage disequilibrium using PLINK with the parameters ‘‘–indep-pairwise 200,250.2’’, and for missing genotype with ‘‘-geno 0.99’’. The unsupervised ADMIXTURE analysis was performed for each value of K, ranging from 2 to 13, and each run was repeated 10 times with different seed values for each repetition (Supplementary Fig. S11). The final results were based on runs with the highest likelihood (K = 7; Supplementary Table S15, Supplementary Figs. S9 and S12). We visualized these results using the PONG software^139^.

### Supplementary Information

Supplementary Information.

Supplementary Tables.

## Data Availability

The *.fastq* files generated from shotgun and enrichment analyses in this study, will be publicly available from the date of publication at the European Nucleotide Archive (ENA) with the accession number PRJEB64776 (https://www.ebi.ac.uk/ena/browser/view/PRJEB64776). All isotopic and anthropological data generated in this study are included in the Supplementary material.

## Code availability

The software used for all analyses is described in detail in the methods section and Supplementary Methods.
