# Supplementary material for: Geographic origin, ancestry, and death circumstances at the Cornaux/Les Sauges Iron Age bridge, Switzerland
Source: Sci Rep. 2024 Jun 17;14:12180. doi: 10.1038/s41598-024-62524-y (PMC11183204; doi:10.1038/s41598-024-62524-y)
Supplement: Supplementary file 1 — Supplementary Information. [file 41598_2024_62524_MOESM1_ESM.pdf]

Geographic origin, ancestry, and death circumstances at the Cornaux/Les Sauges Iron Age bridge, Switzerland.

Zita Laffranchi, Stefania Zingale, Lara Indra, Valentina Coia, Domingo C. Salazar García, Alice Paladin, Marc-Antoine Kaeser, Géraldine Delley, Sönke Szidat, Sandra Lösch, Albert Zink, and Marco Milella

1. Supplementary information: archaeological background and history of research

Cornaux/Les Sauges (henceforth Cornaux) is located in Western Switzerland on the course of the Thielle River at the foot of the eastern slope of the Jura mountains. The archaeological site was discovered during the second Jura Water Correction (1962-1973), a vast undertaking aimed at regulating the level of the lakes of Biel, Murten, and Neuchâtel, mainly by widening and overdrawing the watercourses already developed or canalized during the first Jura Water Correction (1868-1879). Considering the density of finds discovered throughout the region since the middle of the 19th century and the exceptional preservation of archaeological remains in these wetlands (particularly Neolithic and Bronze Age pile-dwellings), this civil engineering operation was accompanied by an ambitious program of archaeological survey and excavations. Under the direction of prehistorian Hanni Schwab (1922-2004), these archaeological operations resulted in the identification and documentation of a considerable number of diverse sites, most of which have been published with significant delay primarily due to inadequate funding for post-excavation analysis.

The site of Cornaux was discovered and excavated from November 2, 1965 to February 26, 1966 by teams composed mainly of construction workers and inexperienced students. The explored area covers a sloping surface of 750 m^2 (25 m wide x 30 m long) on the banks of the Thielle. Difficult conditions (rain, snowstorms, frost, and water infiltrations) required two prolonged interruptions of the fieldwork in December 1965 and January 1966. Deep mud and thick levels of unstable sand meant that the stratigraphic units were under a constant threat of collapse.

The excavation revealed a considerable number of extremely well-preserved architectural timbers distributed rather chaotically in the riverbed. Reconstruction efforts identified these remains as a bridge built according to techniques now well attested in the region for the Iron Age and Roman periods [1, 2]. According to new dendrochronological analyses [3, 4],

this bridge was built around 135 BCE and underwent partial repairs between 120 and 115 BCE, and possibly 105 BCE (this terminus *ante quem* is based on an ornate plate originally interpreted as a cart element, but which could be attributed to the bridge, according to Ramseyer[4]).

The archaeological material fits well into this chronological spectrum. Typologically, the pottery (126 vessels, 75% of which was coarse ware) and the iron and bronze finds (weapons, ornaments, tools, utensils, a coin, etc.) can be dated to the end of the Second Iron Age, precisely to the La Tène D1 phase (150-90 BCE), with the exception of a few older objects (La Tène C) concentrated in upper part of the slope and attributed by H. Schwab to a "habitat" (Schwab[5] also reports some isolated Roman and Neolithic finds, which were well circumscribed in plan and stratigraphy and therefore do not call into question the homogeneity of the whole).

The site also yielded a large number of animal bones (cattle, horses, pigs, goats, sheep, a goose, an otter etc.) as well as human skeletons, both whole and partial, five of which presented the exceptional preservation of brain tissue or organic endocranial remains. Many of these skeletons were intermingled with the beams of the bridge deck and framework (Fig. 1). The zooarchaeological study highlighted that the only animal specimens preserving the anatomical connection were the ones found on the lower bank and the bed of the river: cattle (MNI:3) and horses (MNI: 2). The other domestic species (e.g., pigs and sheep) were represented by fragmentary isolated bones in some case showing traces of cutting and butchering and were localized in the upper part of the slope and related to a possible settlement. The suggested interpretation is that there were only horses and cattle involved in the possible convoy crossing the bridge[6,4]. The arrangement of the finds, the stratigraphy, and the taphonomy of the remains led H. Schwab to a catastrophic interpretation. She concluded that the bridge likely collapsed under the effect of a violent flood carrying a convoy including animals, people, and their goods into the water[5,7].

This interpretation, which guided H. Schwab from the beginning of the excavation, also relied on anthropological and archaeozoological analyses conducted by M.R. Sauter, G. Pilleri, and U. Imhof. It was immediately questioned by many authors [8, 9, 10, 11] because of the indirect implications of this event-driven approach for the understanding of the neighboring (3 km upstream) eponymous site of La Tène[12], which is partially similar in appearance[13, 14].

The recent archaeological re-examination by Denis Ramseyer[4] has generally confirmed the relevance of H. Schwab's interpretation according to sedimentary processes, which were also corroborated by geoarchaeologists Jean-Pierre Garcia & Christophe Petit[15]. However, it remains possible, or even probable, that before the collapse of the bridge around 100 BCE, this crossing point on the Thielle had already hosted "sacrificial" deposits — as evidenced in

particular by the isolated find of a bronze sheet of a sword scabbard methodically folded following documented practices of the ritual mutilation of weapons now firmly attested in the Celtic world in the 3rd and 2nd centuries BCE[16].

The first morphological analysis of the skeletal remains from the site was provided by Sauter[17] and included a first estimate of the minimum number of individuals (20), demographic profiles (17 adults: 12 males, 2 females, and 3 indeterminate and 3 non adults) as well as data on the presence of traumatic lesions, stature, and anthropometric patterns.

2. Supplementary methods: isotopic analyses

Bone collagen extraction and mass spectrometric analyses (Carbon, Nitrogen, Sulfur stable isotopes)

Stable carbon ($\delta^{13}C$) and nitrogen ($\delta^{15}N$) isotope ratios in bone collagen are widely used to reconstruct ancient human diets[18]. $\delta^{13}C$ provides insights about the dietary contribution of C_3 and C_4 plant products[19]. $\delta^{15}N$ values reflect the trophic level of an organism, and the relative intake of animal and plant proteins. Sulfur isotope ratios ($\delta^{34}S$) are influenced by local factors, differentiate between terrestrial and marine environments, and help to assess marine contributions to the diet. $\delta^{34}S$ values in freshwater ecosystems provide insights into dietary habits, and their correlation with local geology suggests their potential use in estimating territorial mobility[20,21].

The extraction of bone collagen was performed following an acid–base–acid extraction method modified after Ambrose [22] [23], DeNiro[24], and Longin[25]. After washing with distilled water, all samples were pulverized in a mix miller at 20 bps for 60 s. Then, 500 mg ± 3 mg of bone powder was demineralized with 10 ml of 1 M hydrochloric acid (HCl) for 20 min at room temperature. The solution was washed until neutral (pH ~ 6–7). About 10 ml of 0.125 M of sodium hydroxide (NaOH) was added and left for incubation at room temperature for 20 h. The solution was then washed until neutral, and 10 ml of 0.001 M HCl was added. The samples were placed in a water bath for incubation at 90°C (10–17 h). The solubilized collagen was filtered (VitraPOR filter-funnel, porosity 16–40 µm) and lyophilized at 0.42 mbar for a minimum of 48 h. Of each sample, three times 3.0 mg ± 0.3 mg collagen was weighed into tin capsules.

The isotope ratios of carbon ($^{13}C/^{12}C$), nitrogen ($^{15}N/^{14}N$), and sulfur ($^{34}S/^{32}S$) were measured by isotope ratio mass spectrometry at Isolab GmbH, Schweitenkirchen, Germany. The average of three measurements per sample was provided and was used for subsequent

analyses. Results are reported in δ-notation in units of per mil (‰) according to the international standards of Vienna Pee Dee Belemnite (VPDB) for carbon, Ambient Inhalable Reservoir (AIR) for nitrogen, and Vienna Canyon Diablo Troilite (VCDT) for Sulfur. In addition, the laboratory internal standards STD R (collagen from cowhide from the EU project TRACE) and for most samples also STD BRA (collagen from Brazilian cowhide) were reported. Internal analytical errors were recorded as ±0.1‰ for $\delta^{13}C$, ±0.2‰ for $\delta^{15}N$, and ±0.3‰ for $\delta^{34}S$ (standard error of the means calculated from 3 or 4 measurements).

We selected samples with a value of >1% collagen portion of dry bone (wt % = amount of extracted collagen/amount of bone powder used for extraction × 100). The molar C: N ratio ([%C/%N] × [14.007/12.011]) in the range of 2.9–3.6 was considered as a good quality collagen indicator[23], same as when % C was in the range of 30%–47% and % N in the range of 11%–17.3%[22, 23, 26]. When at least one of the quality criteria was not within the stated range, we excluded the sample from further evaluation. We considered as good sulfur values when the C: N quality criteria were accepted, and in addition, for mammals and birds %S was within the range of 0.15%–0.35%, the C:S ratio between 300 and 900, and the N:S ratio between 100 and 300, while for archaeological fish %S within the range 0.40-0.85%, the C:S ratio between 175±50 and the N:S ratio between 60±20[27].

DIC samples were prepared for CO_2 release as follows: a portion of sample was injected into 12-ml vials pre-filled with helium and 5 drops of 65% phosphoric acid and agitated in a Vortex mixed for 30 seconds. Afterwards, the vials were left at room temperature for 15 to 36 hours to reach a state of equilibrium[28]. The CO2 was separated from other residual gases by chromatography using a helium carrier gas in a Gas Bench (Thermo Finnigan, Bremen, Germany) system interfaced with a mass spectrometer[29]. Isotopic ratios in DIC samples were analyzed using a Delta XP mass spectrometer (IRMS). Three internal standards of Na_2CO_3 solution have been used (DIC-A, DIC-B and DIC-T), each with a different isotopic composition (-4.9‰, -9.50‰, +28.59‰ vs VPDB). These standards of about 15 L (preserved at room temperature by poisoning with mercuric chloride) were measured with an Elemental Analyzer online with a Delta Plus XL mass spectrometer (IRMS). Additionally, the carbonates were also measured with a Gas Bench (Thermo Finnigan, Bremen, Germany) system interfaced with a mass spectrometer (Delta XP). Precision calculated after correction of the mass spectrometer daily drift and from standards systematically interspersed in analytical batches was better than ± 0.1‰ for $\delta^{13}C$ in DIC.

Radiocarbon analyses

LARA Laboratory

The preparation of the bones followed Szidat et al. [30] and was slightly modified by the implementation of an ultrafiltration step, as recently realized in Steuri et al. [31]. The samples were cleaned by ultrasonication in ultra-pure water and ground to 0.5–1 mm with a ball mill. The chemical treatment included the following steps: 0.5 mol/L hydrochloric acid (HCl) for 60 hr., 0.25 mol/L sodium hydroxide (NaOH) for 1 hr., 0.5 mol/L HCl for 1 hr, followed by a gelatinization in diluted HCl at pH 3 and 60°C overnight. The warm solution was filtered using precleaned Ezee-Filters, ultrafiltration was performed with Vivaspin™ 15 30 kDa molecular weight cut-offs (MWCO) ultrafilters (Sartorius) and the high-molecular-weight fraction was lyophilized. The extracted collagen was combusted and graphitized with an automated graphitization equipment (AGE). The ^{14}C measurements were performed with the accelerator mass spectrometry (AMS) system MICADAS using ^{14}C-free sodium acetate and the NIST standard oxalic acid II (SRM 4990C) for blank subtraction, standard normalization, and correction for isotope fractionations[32].

Tandem Laboratory

The pre-treatment of bone samples followed a modified version of the protocol described in Longin[25]. The surface of bone samples was mechanically cleaned (scraping, in some cases sand blasting) and ultrasonically cleaned in boiled, distilled water (pH 3). The bones were then grinded in a mortar. 0.8M HCl was added stirred (30 min, circa 10°C) (apatite removed). Distilled water kept at pH 3 was added to the insoluble fraction, which was heated while stirring (10 h, 90° C). The fraction to be ^{14}C-dated in the accelerator (early MICADAS machine from the Swiss company IONPLUS) was combusted to CO_2 and graphitized using a Fe-catalyst reaction (see [33] for more details).

Oxygen stable isotope analysis: sample preparation and analysis

The analysis of oxygen isotopic ratios ($^{18}O/^{16}O$, $\delta^{18}O$) is a powerful tool for the reconstruction of past environments. These analyses are usually performed in animal and human tooth enamel with the aim of investigating paleoclimate and paleoseasonality, animal husbandry practices, but also human and animal territorial mobility and geographical provenance[34]. These analyses are also useful in the study of human sociocultural practices such as the process of breastfeeding, weaning, and past culinary practices (e.g., stewing and brewing)

[35]. Oxygen isotopic signature in mammalian tissues reflects the isotopic values of the water ingested (as drinking water or contained in food) during the time of formation of the skeletal tissue. The ingested water often results to be closely in line with the isotopic composition of the local meteoric precipitation, which shows a strong geographical trend, influenced by different variables (e.g., latitude, distance from the coast, altitude, temperature, and humidity) [36, 37].

The $\delta^{18}O$ ratios from dental enamel samples (from second or third molar or first or second premolar) of 10 individuals from the context of Cornaux, as well as from four animals (herbivores and pigs) (Supplementary Table S6), were measured. Samples weighing between 4.5 and 9 mg were chemically treated following protocols originally proposed by [38], and modified by [39, 40, 41]. Enamel powder samples were treated for 4 h in 0.1 M acetic acid [CH3COOH] (0.1 ml solution/0.1 mg of sample), rinsed several times (at least five times) with distilled water and freeze-dried. Isotope measurements were performed on a Thermo Scientific Delta V Plus continuous flow–isotope ratio mass spectrometer (IRMS) equipped with a Gas-Bench II carbonate sample preparation and inlet system in the IsoTOPIK Laboratory at the University of Burgos Scientific and Technology Centre. Carbonate powder samples were digested in He-flushed borosilicate exetainers at 70 °C using water-free 99.9% phosphoric acid. To assess the accuracy (external analytical precision) and to calibrate the obtained isotopic values, the results were measured against IAEA-603 ($\delta^{13}C$ = +2.46‰, $\delta^{18}O$ = -2.37‰) and NBS18 ($\delta^{13}C$ = -5.014‰, $\delta^{18}O$ = -23.2‰) international reference materials. Results are expressed as parts per thousand with respect to the Vienna Pee Dee Belemnite standard (‰ VPDB). The average 1 sigma internal precision error (ten injections per sample) is 0.03‰ for $\delta^{18}O$ and 0.03‰ for $\delta^{13}C$. According to the reference material analyses, stable carbon and oxygen isotope values were measured accurately to the nearest 0.02‰ and 0.02‰, respectively. Reproducibility was checked by duplicate analysis of some samples, and in the case of seemingly aberrant initial results, repeat sampling and analysis was undertaken.

We also analyzed $\delta^{18}O$ from three water samples, two from the river Thielle and one from the close Lake Neuchâtel (in correspondence with the beach of La Tène) (Supplementary Table S6). The isotopic analyses of the water samples were carried out by injecting 1.8 microliters into a Picarro L-2140i. The replications of internal standards (contrasted with IAEA international standards) indicate errors of less than 0.1‰ and 0.5‰ for $\delta^{18}O$ and δD, respectively. To approach geographical localization of the samples, we also converted carbonate $\delta^{18}O$ values (VPDB) to the oxygen isotopic composition of drinking water ($\delta^{18}O_{DW}$ vs. VSMOW) using the equation provided by Chenery et al. [42].

The Kernel density estimation and the calculation of the 95% density region of human oxygen values were calculated with the function hdr.den in the R package hdrcde[43]. The optimal bandwidth for the kernel density estimation was selected using the method of Sheather and Jones[44], with function *bw.SJ* in R (version 4.3.2).

Strontium isotope analysis: sample preparation and analysis

Strontium isotopic analysis ($^{87}Sr/^{86}Sr$) of skeletal material is commonly used to detect geographic provenance and mobility among mammals, including humans [45,46]. The tooth enamel records the isotopic signal of when it was formed during the earliest stages of life, whereas the bone isotopic signal reflects a period closer to the time of death of the individual[47] Since the radiogenic isotope ^{87}Sr forms by radioactive decay from rubidium (^{87}Rb), the $^{87}Sr/^{86}Sr$ signature of a specific location is determined by the underlying bedrock age and its content of Rb[48]. A specific geological strontium signature is incorporated into the hard body tissues by direct substitution for calcium[49] since strontium enters the ecosystem without fractionation[50].

The $^{87}Sr/^{86}Sr$ ratios from dental enamel samples (from second or third molar or first or second premolar) of ten individuals, as well as from four animals (herbivores and pigs) from Cornaux (Supplementary Table S5), four modern plants and four land snail shells from Rüfenacht (Bern) were measured. Sampling and the first preparation were carried out at the ANTARQBIO lab of the Universitat de València (Spain), while the chemical sample preparation and analysis were done in dedicated isotope facilities of the University of Cape Town (South Africa), as described below. Archaeological teeth, plants and snail shells were brought directly to the ANTARQBIO lab for their sampling and cleaning. The pretreatment of plant (all leaves) and land snail shell samples was realized following the procedures described in Copeland et al.[51] and Wong et al.[52]. The preparation included the cleaning, drying, incineration (at 500° C for 8 hours) and weighing (circa 20 mg of ash) for the plants and a first mechanic cleaning followed by a rinse with acetone and three more rinses with Milli-Q for 20 min each in the ultrasonic bath then the drying and weighing (~10-30 mg) for the snail shells. The cleaned enamel samples (ca. 20 mg for each type of material) were digested with 2mL bi-distilled 65% HNO_3 in a closed Teflon beaker placed on a hotplate at 140 °C for an hour. Digested samples were then dried and redissolved in 1.5 mL of bi-distilled 2M HNO_3. These re-dissolved samples were centrifuged at 4000 rpm for 20 minutes, and the supernatant was collected for strontium separation chemistry. A separate fraction for each sample in this step

was used to calculate the concentration with ^{88}Sr intensity (V) regression equation built with SRM987 standard from NIST (National Institute of Standards and Technology, Gaithersburg, MD, USA). Strontium was then isolated with 200µl of Eichrom Sr.Spec resin loaded in Bio-Spin Disposable Chromatography Bio-Rad Columns following the method of Pin et al. [53]. The separated strontium fraction for each sample was dried down, dissolved in 2 ml 0.2% bi-distilled HNO_3 and diluted to 200 ppb Sr concentrations for isotope analysis.

$^{87}Sr/^{86}Sr$ ratios were measured using a NuPlasma HR multicollector inductively-coupled-plasma mass spectrometer (MC-ICP-MS). Sample analyses were referenced to bracketing analyses of SRM987, using a $^{87}Sr/^{86}Sr$ reference value of 0.710255 from NIST987. All strontium isotope data are corrected for isobaric rubidium interference at 87 amu using the measured signal for ^{85}Rb and the natural $^{85}Rb/^{87}Rb$ ratio. Instrumental mass fractionation was corrected using the measured $^{86}Sr/^{88}Sr$ ratio and the exponential law, and a true $^{86}Sr/^{88}Sr$ value of 0.1194[51]. Results for repeat analyses of an in-house carbonate standard NM95 ($^{87}Sr/^{86}Sr$ = 0.708900; 2 sigma 0.000034; n=14) and an in-house ashed plant standard NAMISO316 ($^{87}Sr/^{86}Sr$ = 0.720517; 2 sigma 0.000019; n=6) processed and measured with the batches of samples in this study are in agreement with long-term results for these two in-house standards ($^{87}Sr/^{86}Sr$; 0.708911; 2 sigma 0.000039; n=545) ($^{87}Sr/^{86}Sr$; 0.72051; 2 sigma 0.000043; n=8). For every two batches one blank was added to assess the cleanness of the process; there was no peak and, thus, no contamination from external Sr in any of the batches.

3. Supplementary methods: paleogenetic analyses

Samples selection and sampling.

Based on the preservation and availability of the petrous portion of the temporal bone (PP) and ear ossicles, eleven individuals from Cornaux were selected for ancient DNA (aDNA) analysis. Therefore, we used nine PP, three still articulated to the other cranial bones [COR-2 (3436), COR-3 (3430), and COR-19 (3437)] and six being detached [COR-5 (3435), COR-7 (3433), COR-8 (3432), COR-11 (3431), COR-15/17 (3429), and COR-21 (3438)]. In addition, the ear ossicles were used in two cases [COR-9 (3434) and COR-16 (3439)] (Supplementary Table S7).

Sampling was performed under clean conditions at the Laboratory of the Department of Physical Anthropology, Institute of Forensic Medicine, University of Bern, Switzerland. Before sampling, the surface of the bones was cleaned using a low-concentration H_2O_2 solution (3%) and then ddH_2O. For the PP we generated bone powder from the petrous pyramid using a driller

after removing the outer layer (approx. 200 mg for each sample), and whole middle ear bones (69 – 81 mg) were collected and stored for DNA extraction.
Further laboratory work was performed in a dedicated pre-PCR area of the aDNA laboratory at the Institute for Mummy Studies, Eurac Research, Bolzano, Italy, following all the strict rules required for aDNA analyses.

DNA Extraction

A silica membrane-based method was used for DNA extraction following a modified protocol based on[54, 55]. The powder obtained from the sampling of the PP and the middle ear bones as a whole were dissolved in EDTA extraction buffer (0.5 M EDTA pH8, 20 mg/ml proteinase K) at 40° C overnight. After incubation, the extract was centrifuged at 5000 x g for 2 minutes to separate undissolved matter from the supernatant solution. The supernatant was then transferred into 4 ml Amicons (Ultra-4 centrifugal filter unit (30 kDa), Merck Millipore) and centrifuged at 2500 x g until reaching a final supernatant volume of 100 µL. The DNA concentrate was purified using the MinElute PCR Purification Kit (Qiagen) and eluted using EB buffer[56]. The purified DNA samples and the extraction blank control were quantified using the QuantiFluor® ONE dsDNA system (Promega[57]) and stored at -20°C until library preparation.

Library Preparation

The extracted DNA was converted into double-stranded, double-indexed Illumina libraries for sequencing using a modified protocol according to Meyer & Kircher[58]. For library preparation, 25µl of DNA extracts were used and blank controls were included in every step, comprised of amplification.
Blunt-end repair of the DNA extract was carried out by combining the DNA extract with a 45 µl reaction mix, which consisted of NEBNext® End repair buffer (New England BioLabs Inc.), NEBNext® End repair enzyme mix (New England BioLabs Inc.), 10 mg/ml BSA (New England BioLabs Inc.), and ddH_2O. The reaction mixture was incubated for 15 minutes at 25 °C, followed by 5 minutes at 12 °C. Subsequently, the reaction underwent purification using the MinElute PCR Purification Kit (Qiagen[56]). For the adapter ligation step, a reaction was prepared containing 10X T4 DNA Ligase buffer, PEG 4000 (50%), Adapter Mix (5 µM each), T4 DNA Ligase (5 U/µl), and ddH_2O. This reaction was incubated at 22° C for 20 minutes. After this step, another MinElute purification was performed[56], and the elution volume was

adjusted to 20 µl. To facilitate the fill-in of nicks on the unphosphorylated 3' ends of DNA fragments, the eluted samples (20 µl) were combined with a 20 µl reaction mix containing 10 X Thermopol reaction Buffer (New England BioLabs Inc.), dNTPs (2.5 µM each), and 4 U/µl Bst polymerase large fragment (New England BioLabs Inc.). The reaction was incubated for 20 minutes at 37 °C, followed by 20 minutes at 80 °C. For multiplexed sequencing, sample-specific indexing primers were added to each library through amplification. The amplification reactions had a total volume of 25 µl, with 3 µl of DNA library, NEBNext® Q5U ® Master Mix (New England BioLabs Inc.), BSA (New England BioLabs Inc.), and 10 µM of P5-primer and 5 µM of P7-primer. To increase library complexity and to reduce stacking, three amplification reactions with the same indexing primer were performed for each sample. The thermal profile started with 2 minutes at 95 °C, followed by 12 cycles with 30 seconds at 95 °C, 30 seconds at 58 °C, and 1 minute at 72 °C. The final extension step lasted for 10 minutes at 72 °C. After pooling the amplified libraries, MinElute purification was performed[56], and the DNA was eluted in 25 µl EB-Buffer. Purified libraries were subsequently quantified using a High Sensitivity Bioanalyzer Kit (Agilent Technologies[59]) and a QuantiFluor® ONE dsDNA System by Promega[57].

Molecular Screening and In-Solution Target Enrichment

The indexed libraries were shotgun sequenced at the facility Macrogen (Seoul) on a HiseqX platform (150 bp PE) (Supplementary Table S8). All eleven samples fulfilled the quality criteria (presence of damage pattern of aDNA and content of human endogenous DNA ≥ 1%) and were then enriched for human DNA using the "Twist Ancient DNA" – kit (Twist Bioscience) with a modified protocol optimized for aDNA[60]. The procedure was additionally adjusted by reducing the cycle numbers for post-enrichment amplification from 23 to 15. The reagent includes 1 434 155 probes targeting 1 352 535 SNPs of the human genome (including among others the core 1 240k SNPs, 81 925 on the Y-Chromosome, and 94 586 phenotypically relevant targets)[61]. For this study, the mitochondrial panel from Twist was spiked in during the experiment.

The library quantity input for the target enrichment ranged between 356.5 – 1300 ng, to reach this quantity, adapted libraries were amplified several times and then pooled and dried at 30 °C using a vacuum concentrator (Eppendorf). For the hybridization, two different reactions were prepared: The probe solution contains the Twist Hybridization Mix, the Twist Custom Panel – AncientDNA_1.41M SNP, and the Twist Mitochondrial Panel, and the blocker solution is composed of the Twist Blocker Solution and the Twist Universal Blockers which are added to

the dried indexed samples for resuspending. The probe solution is heated to 95°C for 2 minutes and then immediately cooled on ice for 5 minutes. Meanwhile, the blocker solution containing the resuspended indexed library pool is heated at 95°C for 5 minutes. Both the probe solution and resuspended indexed library pool are equilibrated at room temperature for 5 minutes. After mixing the two solutions, we added the Twist Hybridization Enhancer on top of the entire capture reaction. The hybridization reaction was incubated at 62°C for 16 hours in a thermal cycler and was then transferred to the Twist Streptavidin Binding Beads and Binding Buffer mix and incubated on a shaker at room temperature for 30 minutes in order to bind the targets to the beads. The mix of Streptavidin Binding beads and hybridization reaction was washed in several steps with the Twist Wash Buffer 1 (at room temperature) and Twist Wash Buffer 2 (at 48 °C), removing in between the supernatant by pelleting the beads on a magnetic particle collector, in order to remove the non-target DNA. After the last washing step and removal of the supernatant, ddH_2O was added to the beads, and the Streptavidin Binding Bead slurry was incubated on ice until settled. Half of the Streptavidin Binding Bead slurry was used for post-enrichment amplification using the Twist Equinox Library Amp Mix and the Twist Amplification Primers (10 uM) following initialization at 98°C for 45 seconds, then 15 cycles of 98°C for 15 seconds, 60°C for 30 seconds, and 72°C for 30 seconds, followed by a final extension at 72°C for 1 minute. The amplified enriched libraries were then purified using the Twist DNA Purification Beads and 80% freshly prepared ethanol and then resuspended in EB buffer. Subsequent quantification of the enriched libraries, they were sequenced again on an Illumina Hiseq X (150 bp PE) system at the Macrogen Sequencing Centre (Seoul).

Bioinformatic analyses and aDNA authentication

A total of 703070376 (PE) new DNA sequences were generated for this study via shotgun sequencing and a total of 620278854 (PE) were generated via sequencing of enriched libraries. The sequencing reads were trimmed for removing adapters and merged following specific quality criteria: the number of overlapping bases of 11, and the minimum length of the assembled sequences of 25[62]. After merging the sequencing reads, alignment to the Human Reference Genome (GRCh37/hg19) and the revised Cambridge Reference Sequence (rCRS) was performed using BWA (v. 0.7.17) setting the minimum mapping quality to 30[63]. With DeDup (v. 0.12.8), reads with the same start and end and the same orientation (duplicates) were removed[64]. The ancient reads were examined for damage patterns, including fragmentation and misincorporation, using the software mapDamage (v. 2.2.1)[65]. Exogenous human contamination was estimated at both nuclear and mitochondrial levels. For all samples, the

software Schmutzi[66] was used to infer contamination levels on the mtDNA. For male individuals only, the method implemented in ANGSD (Analysis of Next Generation Sequencing Data) was applied to estimate contamination based on X-Chromosome data[67]. For this analysis, we excluded the pseudoautosomal region of the X chromosome and used a minimum base and mapping quality threshold of 30 with a coverage filter of 2. Thresholds for mtDNA contamination and nuclear contamination are ≤ 5% and ≤ 3% respectively. The results of both methods are reported in Supplementary Table S11.

For the two samples that show modern human contamination, we applied PMDtools with a quality threshold of 30. This software utilizes an approach that assesses aDNA damage patterns incorporating postmortem damage (PMD), base quality scores, and biological polymorphism in order to distinguish degraded DNA sequences that are unlikely to originate from modern contamination. This method assigns to each sequence a PMD score, with positive values indicating strong evidence that the sequence is indeed of ancient origin[68]. Data generated from shotgun sequencing and enrichment that met the set quality criteria, were merged for each individual, and merged data was used for downstream analyses in which BAM files after quality filter q30 were used. In Table S8 we summarize quality statistics for shotgun data, and in Supplementary Table S9 the ones from merged data.

Sex determination and unilinear transmitted markers

Biological sex was determined by calculating the ratio of sequences aligning to the X and Y Chromosomes as reported in[69]. We conducted a second verification using the method described in Mittnik et al.[70]. We applied these methods to all 10 samples since those filtered by PMD-tools retained a sufficient number of human reads requested by the two methods (100000 and 1000 respectively) for the accurate performance of the genetic sex determination (Supplementary Table S9).

To determine the mtDNA haplogroups, we used reads mapped to the reference mitochondrial genome (rCRS) from the consensus file generated by Schmutzi in 6 samples. For two samples we extracted a vcf file from the rescaled bam file generated by MapDamage. To ensure the accuracy of the haplogroup assignment, we established a threshold of a mean coverage of the mtDNA genome of 3 X (Supplementary Table S11). Since the two contaminated samples did not reach the set threshold after PMDtools filtering, we included in the results only eight samples from Cornaux. We exported the generated files and used them for the assignment in HaploGrep3, an automated mitochondrial haplogroup assignment tool

available via https://haplogrep.i-med.ac.at/ (based on PhyloTree; mtDNA tree build 17, available via www.phylotree.org/)[71].

To identify the Y-Chromosomal haplogroups of male individuals, we used reads that mapped to the Y-Chromosome SNPs (human reference genome hg19) with a base quality ≥ 30. Using the Yleaf software we assigned the Y-Chromosomal haplogroups[72]. To ensure accuracy, we verified any mutations found in the VCF files against the most recent phylogenetic tree of the human Y-Chromosome (ISOGG; Version: 15.73 Date: 11 July 2020).

Kinship analyses

For evaluating the genetic relatedness among individuals in Cornaux, we applied three different methods based on autosomal markers. The READ method (Relationship Estimation from Ancient DNA[73] and the TKGWV2 method (Thomas Kent Genome-Wide Variants 2[74] were used to infer relatedness up to the 2nd degree. The KIN[75] method can infer genetic relationships up to 3rd degree.

READ calculates and normalizes a mismatch rate across the genome to determine the degree of relationship between pairs of individuals. By employing genotype likelihoods and allele frequencies, READ estimates the probability of sharing zero, one, or two alleles that are identical by descent. This method requires a mean coverage of human reads of at least 0.1 X. TKGWV2 can infer 1st- and 2nd-degree relatedness with as little as 0.026 X average coverage and uses genotype likelihoods and population allele frequencies of genome-wide variants present in the 1000 Genomes Project Phase 3. KIN, instead, uses a Hidden-Markov-Model-based approach to identify biological kinship up to 3rd-degree using at least 0.05 X sequence coverage. Additionally, it allows distinguishing between sibling and parent-child relationships. To further verify and ascertain additional degrees of relatedness, we considered information from mtDNA haplotypes and Y-Chromosomal haplogroups, in conjunction with archaeological, chronological, and anthropological data.

Comparative Analyses and Dataset

For genome-wide downstream analyses, we used merged (shotgun + enrichment) data from eight non-related samples that cover a minimum of 50000 SNPs on the 1240k dataset. We genotyped the Cornaux samples at each SNP using samtools mpileup[76], with a minimum base and mapping quality threshold of 30. To reconstruct pseudo-haploid genotypes, the pileupCaller tool was applied. This tool, available at https://github.com/stschiff/sequenceTools , randomly

assigns alleles between bases at each site. Transitions sites were excluded to minimize errors caused by postmortem damage.

The genotyped samples were merged with previously published genomes from the dataset of David Reich's lab[77], which include data from ancient and present-day individuals from either shotgun sequencing data or in-solution target capture, with a range of coverages. Duplicate entries and potentially related individuals were removed from the dataset. To ensure high-quality data for analysis, only individuals classified as 'PASS' with a minimum coverage of 0.01 and more than 10000 SNPs were retained. We created a dataset (referred as HO-dataset) that includes 473 ancient individuals together with 1575 present-day individuals from worldwide populations genotyped on the Human Origins Array (597573 SNPs) (Supplementary Tables S12 and S13). The ancient set includes newly reported individuals from Cornaux and individuals that had previously been published [78-110] from the same time period (LIA) from several regions from Europe, and ancient individuals that represent major European prehistorical ancestral groups (Western Hunter-Gatherers, Neolithic Anatolian farmers, Neolithic Iranian, and Early Bronze Age Yamnaya group from the Russian steppe [98,111]). We kept 110416 sites after intersecting autosomal SNPs.

A Principal Component Analysis (PCA) was performed to examine the genetic relationships between the studied individuals and other present-day populations and already published ancient individuals from Europe. After merging the samples of Cornaux with the previously described HO_dataset, the determination of overlapping SNPs between the studied samples and the AADR HO_dataset, smartpca from the EIGENSOFT package (version: 16 000) was applied. The lsqproject and shrinkmode parameters were set to YES[112]. The PCA performed with only present-day individuals from Europe (Supplementary Fig. S13, Supplementary Table S12) shows that some individuals from Cornaux have a genetic affinity to populations from Western (France; samples COR-8, COR-9, and COR-16) and Southwestern Europe (Spain; COR-21) while others (COR-2, COR-15/17, COR-5, and COR-11) held intermediate positions between these two main groups.

4. Supplementary Figures

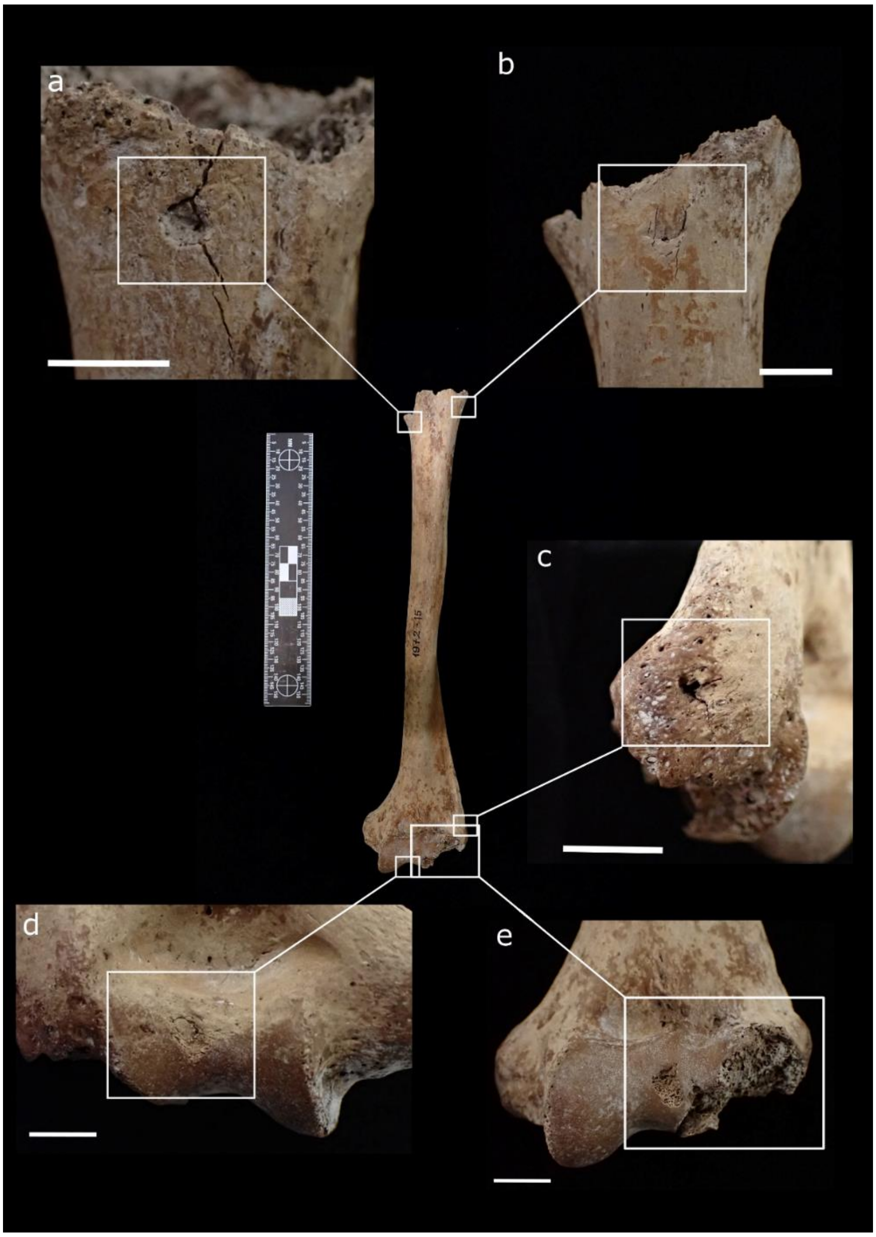

Figure S1. Vertebrate scavenging at Cornaux/Les Sauges. COR-10, left humerus: puncture marks on medial (a) and lateral (b) sides of the proximal shaft, lateral side of distal end (c), and pits on posterior (d) and anterior (e) surface of trochlea.

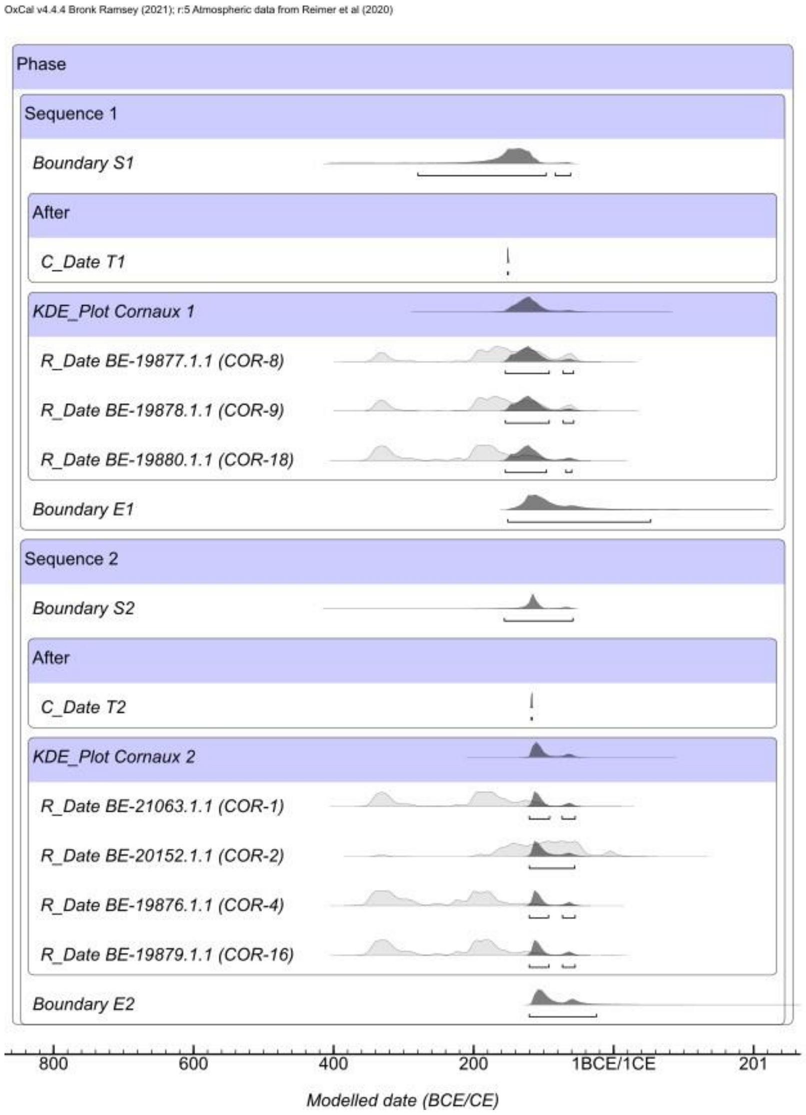

Name	Unmodelled (BCE/CE)		Modelled (BCE/CE)		Indices A_{model}=23.9 $A_{overall}$=22.6					Controls		
	from_95.4	to_95.4	from_95.4	to_95.4	A_{comb}	A	L	P	C	Select	Page	Edit
			Warning! Poor agreement - A= 22.6%(A'c= 60.0%) Warning! Poor agreement - A= 23.9%(A'c= 60.0%)									
▼ Phase										2		
▼ Sequence 1										3		
Boundary S1			-280	-62					52.6	4		
▼ After	-150	...								5		
C_Date T1	-152	-151	-152	-151		100			100	6		
▼ KDE_Plot Cornaux 1									71.2	7		
N Cornaux 1_Kernel	-2	2	-2.01	2		100			99.9	8		
U Cornaux 1_Scale	9.97466e-18	1	0.522	1		100			99.9	9		
R_Date BE-19877.1.1	-346	-53	-155	-58		103.4			80.3	10		
R_Date BE-19878.1.1	-349	-53	-155	-58		89.1			80.9	11		
R_Date BE-19880.1.1	-353	-58	-155	-61		42.8			82	12		
			Warning! Poor agreement - A= 42.8%(A'c= 60.0%)									
Boundary E1			-152	54					71.4	13		
▼ Sequence 2										14		
Boundary S2			-157	-59					88.9	15		
▼ After	-116	...								16		
C_Date T2	-118	-117	-118	-117		100			100	17		
▼ KDE_Plot Cornaux 2									94.2	18		
N Cornaux 2_Kernel	-2	2	-2	2.01		100			99.9	19		
U Cornaux 2_Scale	9.97466e-18	1	0.39	1		100			99.9	20		
R_Date BE-21063.1.1	-353	-57	-121	-56		39.7			93.9	21		
			Warning! Poor agreement - A= 39.7%(A'c= 60.0%)									
R_Date BE-20152.1.1	-195	5	-121	-56		113.2			92.5	22		
R_Date BE-19876.1.1	-358	-108	-121	-56		13.1			94.2	23		
			Warning! Poor agreement - A= 13.1%(A'c= 60.0%)									
R_Date BE-19879.1.1	-355	-60	-121	-56		20.3			94.1	24		
			Warning! Poor agreement - A= 20.3%(A'c= 60.0%)									
Boundary E2			-121	-25					85.5	25		

Figure S2. Plot (left) and output (right) of an Oxcal model including only the skeletons associated to wood beams analyzed dendrochronologically. The model considers two sequences, each one defined by a terminus post quem corresponding to the dendrochronological estimates for the beams.

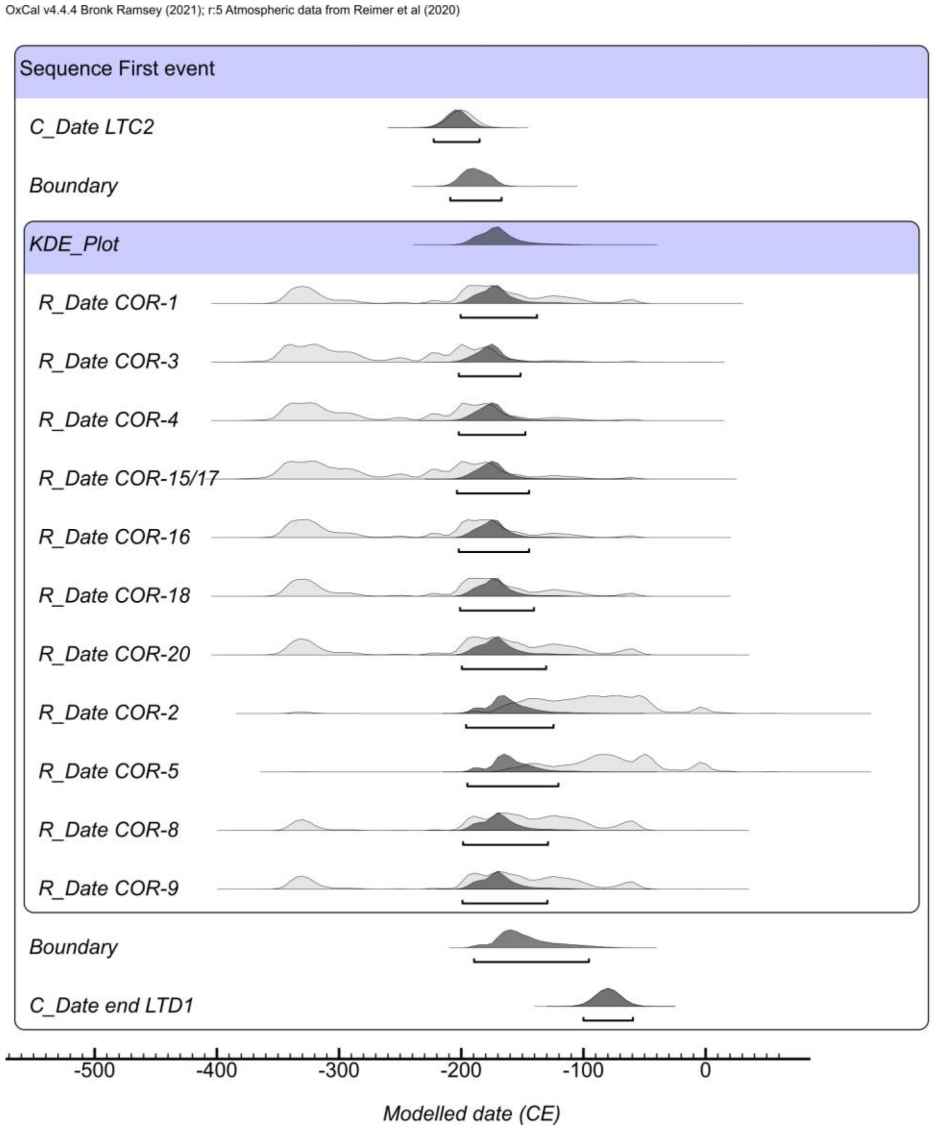

Name		Unmodelled (G)		Modelled (G)		Indices A_{model}=95.9 $A_{overall}$=98					Controls		
		from_95.4	to_95.4	from_95.4	to_95.4	A_{comb}	A	L	P	C	Select	Page	Edit
▼ Sequence First event											☑ 2	☐	
C_Date LTC2		-220	-180	-223	-185		101.9			99.1	☑ 3	☐	
Boundary				-209	-166.5					98.7	☑ 4	☐	
▼ KDE_Plot										99.8	☑ 5	☐	
N _Kernel		-2	2	-2.03	1.99		100.4			96.7	☑ 6	☐	
U _Scale		9.97466e-18	1	0.337	1		100			96	☑ 7	☐	
R_Date COR-1		-351.5	-55	-200	-136		129.7			99.7	☑ 8	☐	
R_Date COR-3		-359	-150	-202.5	-151		92.2			99.7	☑ 9	☐	
R_Date COR-4		-356.5	-106.5	-202.5	-147		107.8			99.6	☑ 10	☐	
R_Date COR-15/17		-359	-106.5	-203.5	-143.5		104.8			99.7	☑ 11	☐	
R_Date COR-16		-353.5	-58.5	-202	-144		117.9			99.7	☑ 12	☐	
R_Date COR-18		-351.5	-56.5	-201	-139.5		126.3			99.6	☑ 13	☐	
R_Date COR-20		-349.5	-52	-199.5	-130		134.7			99.7	☑ 14	☐	
R_Date COR-2		-193	5.5	-196	-124		59.5			99.2	☑ 15	☐	
				Warning! Poor agreement - A= 59.5%(A'c= 60.0%)									
R_Date COR-5		-165	7	-195	-120.5		33.4			99.2	☑ 16	☐	
				Warning! Poor agreement - A= 33.4%(A'c= 60.0%)									
R_Date COR-8		-344	-51	-198.5	-128.5		125.9			99.7	☑ 17	☐	
R_Date COR-9		-347	-51	-199	-129		132.5			99.6	☑ 18	☐	
Boundary				-189	-95.5					97.2	☑ 19	☐	
C_Date end LTD1		-100	-60	-100	-60		100.6			99.1	☑ 20	☐	

Figure S3. Plot (left) and output (right) of an Oxcal model with all radiocarbon dates and a single phase constrained by the estimated chronological extremes of La Tène C2- D1 (ca. 200-80 BCE). The boundaries consider an error of ±10 years

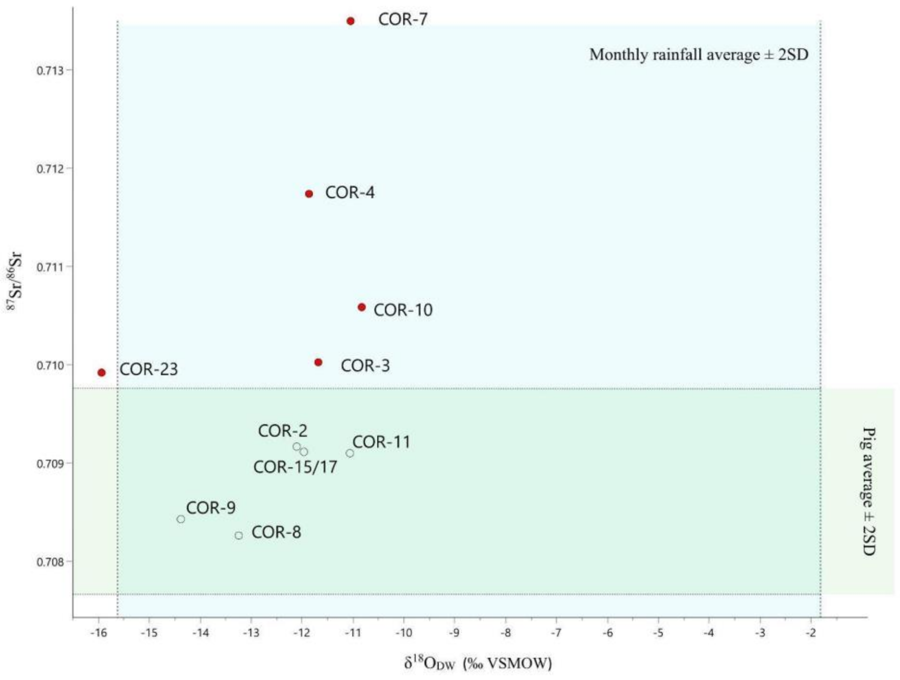

Figure S4. Human oxygen and strontium isotopic ratios. Blue and green areas delimit the local isotopic baseline. Outliers for at least one isotopic system are highlighted in red. See also Table 1.

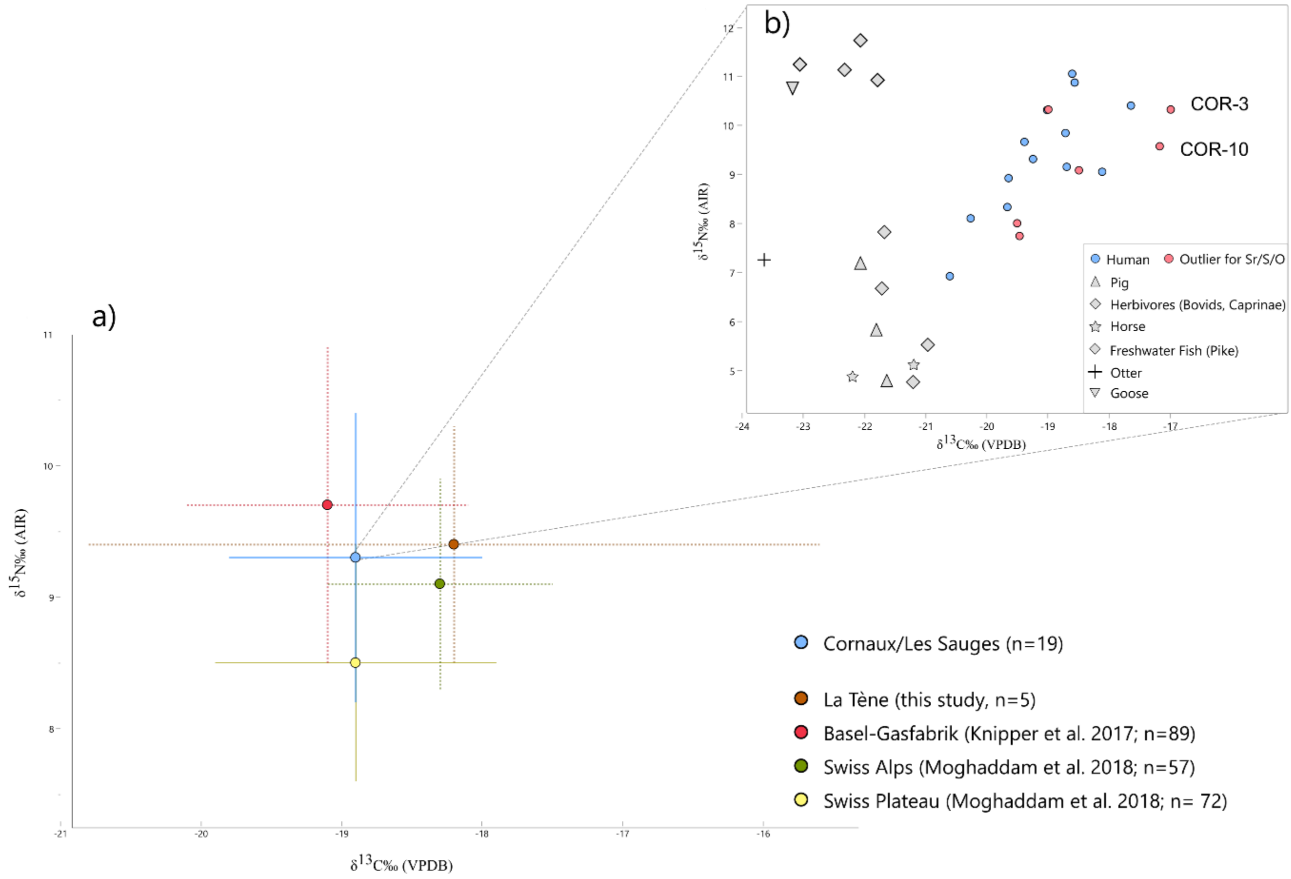

Figure S5. $\delta^{13}C$ and $\delta^{15}N$ values at Cornaux/Les Sauges. (a) Comparison of Cornaux with other late Iron Age Swiss contexts; (b) human and faunal isotopic values from Cornaux. Proxies of freshwater fish are pikes from Auvernier/La Saunerie (Lake Neuchâtel, Neolithic-Bronze Age). Red dots indicate individuals that resulted outliers for $^{87}Sr/^{86}Sr$, $\delta^{18}O$ and/or $\delta^{34}S$. The two isotopic outliers COR-3 and COR-10 are highlighted due to their highest $\delta^{13}C$ values.

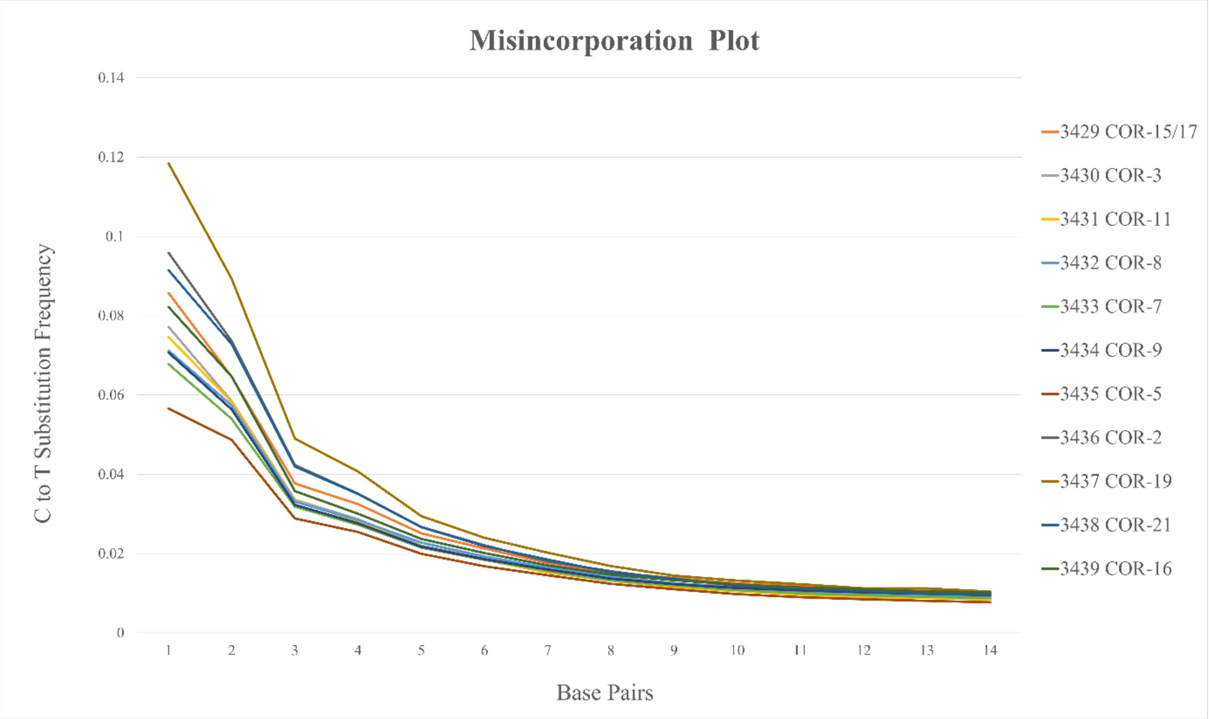

Figure S6. Overview of the misincorporation pattern at the end of the DNA fragments. The deamination damage pattern suggests the authenticity of the ancient DNA of all samples from Cornaux

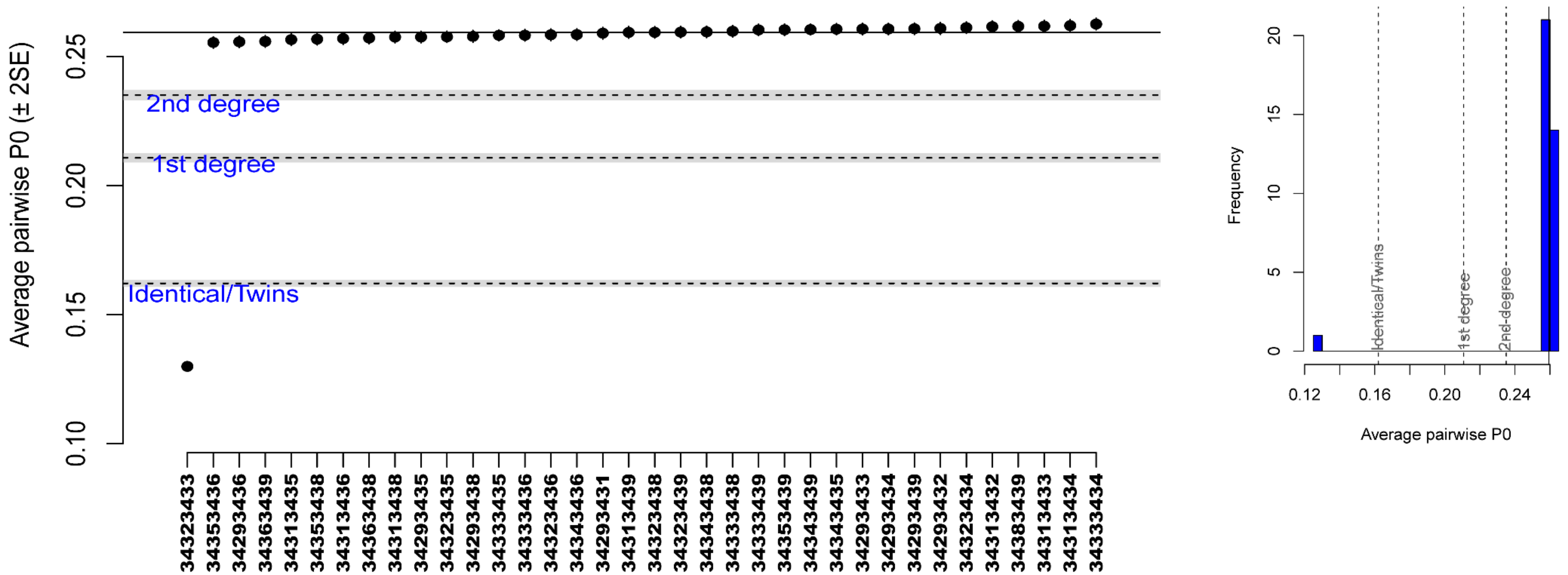

Figure S7 READ results of the pairwise comparisons of 9 individuals from Cornaux. The error bars show two standard errors of the mean (2SE) and the dashed lines show the cutoffs used to classify the related individuals with the gray areas indicating 95% confidence intervals for the cutoffs. The lowest dotted line represents expected values for twins, the second for 1st-degree and the third for 2nd-degree relatives.

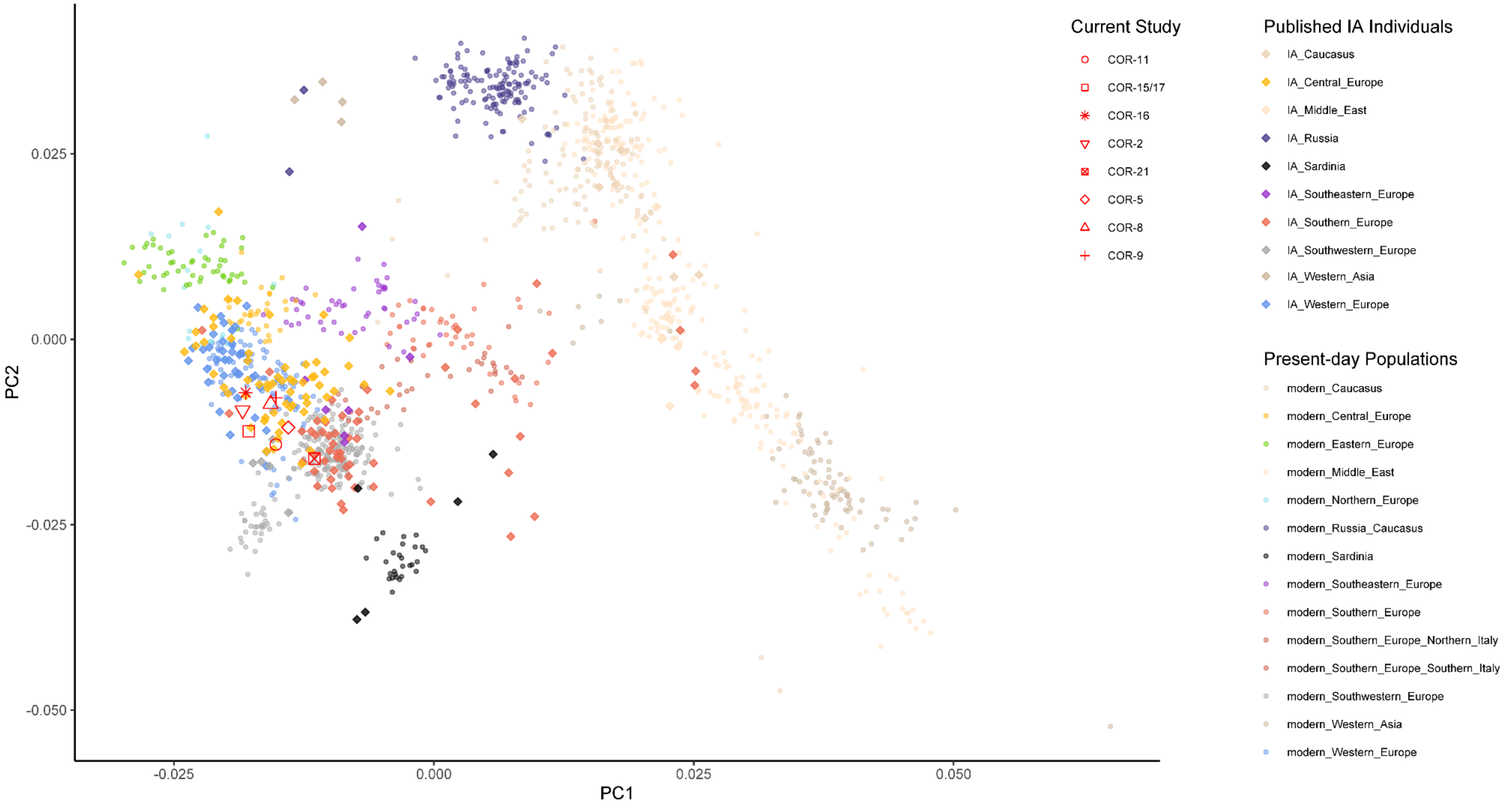

Fig. S8. Principal component analysis of ancient samples from Cornaux and other published ancient individuals from the Iron Age (IA) projected onto the genetic complexity of present-day populations of western Eurasia. Individuals from Cornaux are represented in red symbols whereas circles are used for present-day individuals and diamond-shaped symbols represent published ancient IA individuals from Europe. Different colors indicate the geographic origin.

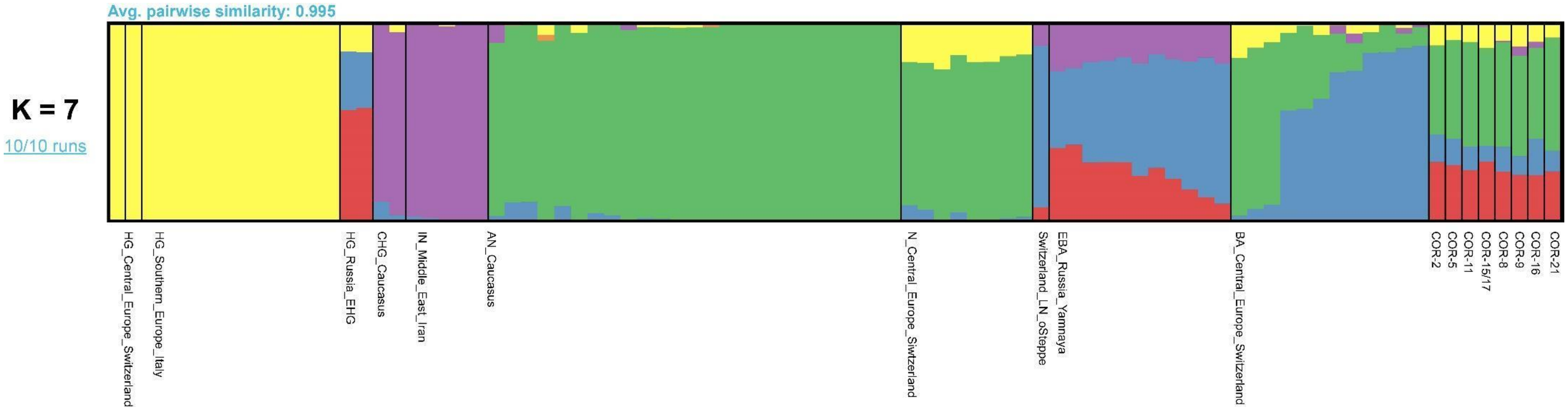

Figure S9. Results of unsupervised clustering analysis (ADMIXTURE; K=7) of the Cornaux samples. On the left, ancient samples are shown that represent the different main ancestry components with different colors: the Western-Hunter-Gatherer component (WHG - here from Mesolithic individuals from central Europe – Switzerland and southern Europe – Italy) is represented in yellow. The Eastern Hunter-Gatherer Component (EHG - from Mesolithic individuals from Russia) is depicted by a combination of yellow, red, and blue colors. The Hunter-Gatherer component from Mesolithic individuals from the Caucasus and the Neolithic-related component, represented by Neolithic Individuals from Iran, are shown in purple. The green color displays the Neolithic-related component from Anatolia (here from Neolithic individuals from Turkey). Finally, the Steppe-related component (from Early Bronze Age – EBA – individuals from Russia; Yamnaya) is indicated by a mix of red-blue and purple colors. We also represent here published individuals from Neolithic and Bronze Age (BA) – Switzerland. The right side displays the Cornaux individuals that show a similar distribution of the different ancestry components, with minor differences (e.g. presence of the purple color).

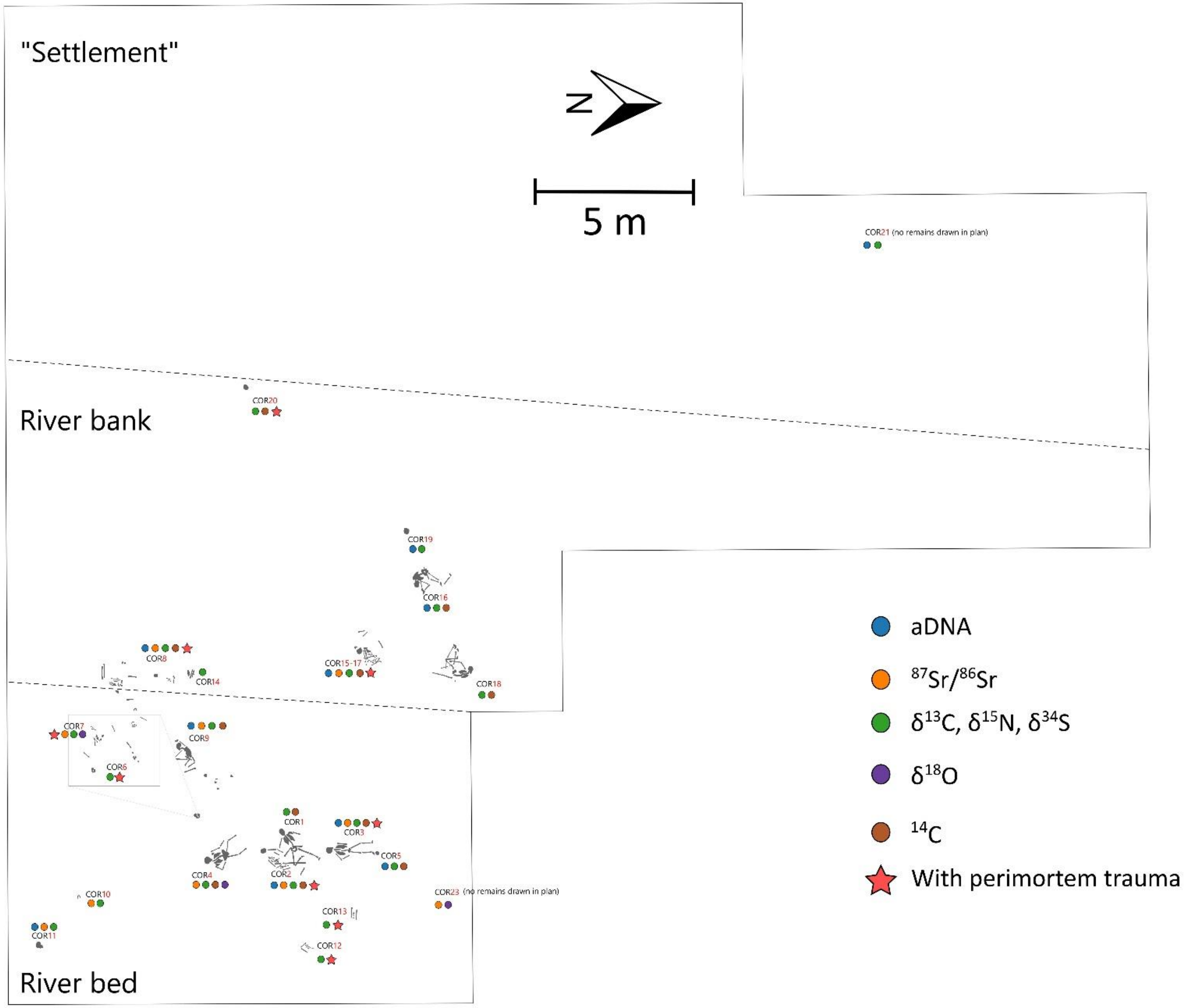

Fig. S10. Digitization of the original archaeological plan showing the spatial distribution of the remains and the range of analyses performed on each individual. The original plan indicates only the position of COR-21 and COR-23 with no drawing of the remains.

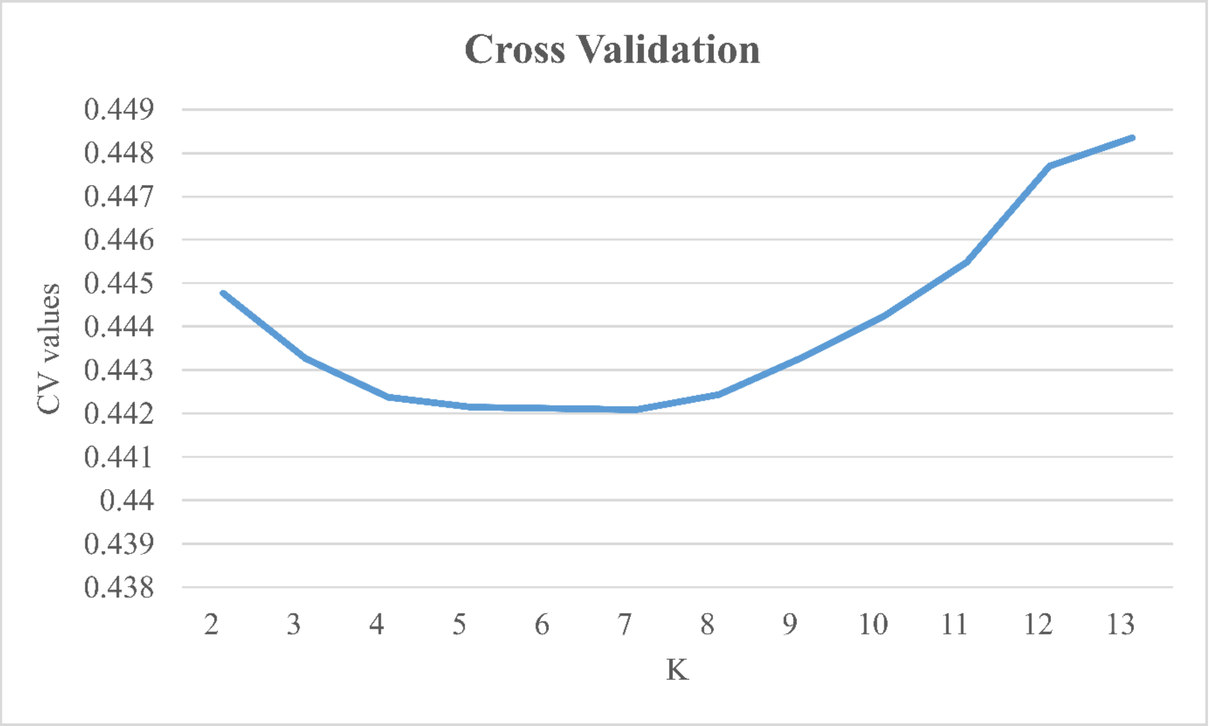

Fig. S11. Cross-Validation (cv) plot with values for unsupervised clustering analyses by ADMIXTURE (K=2 to K=13). The lowest cv value indicates the K with the lowest error.

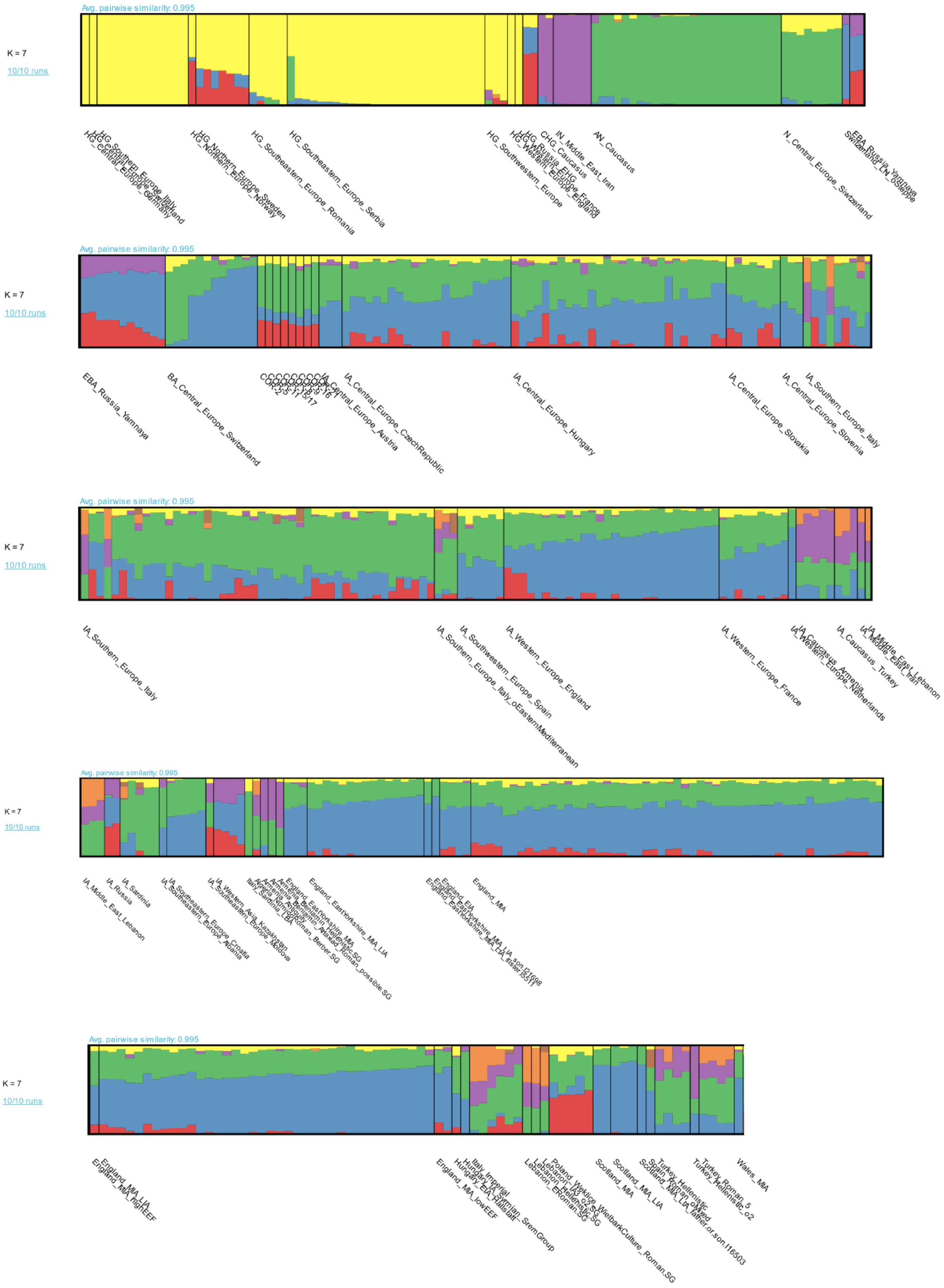

Fig. S12. Results of unsupervised clustering analysis (ADMIXTURE; K=7) of all ancient samples used for this analysis.

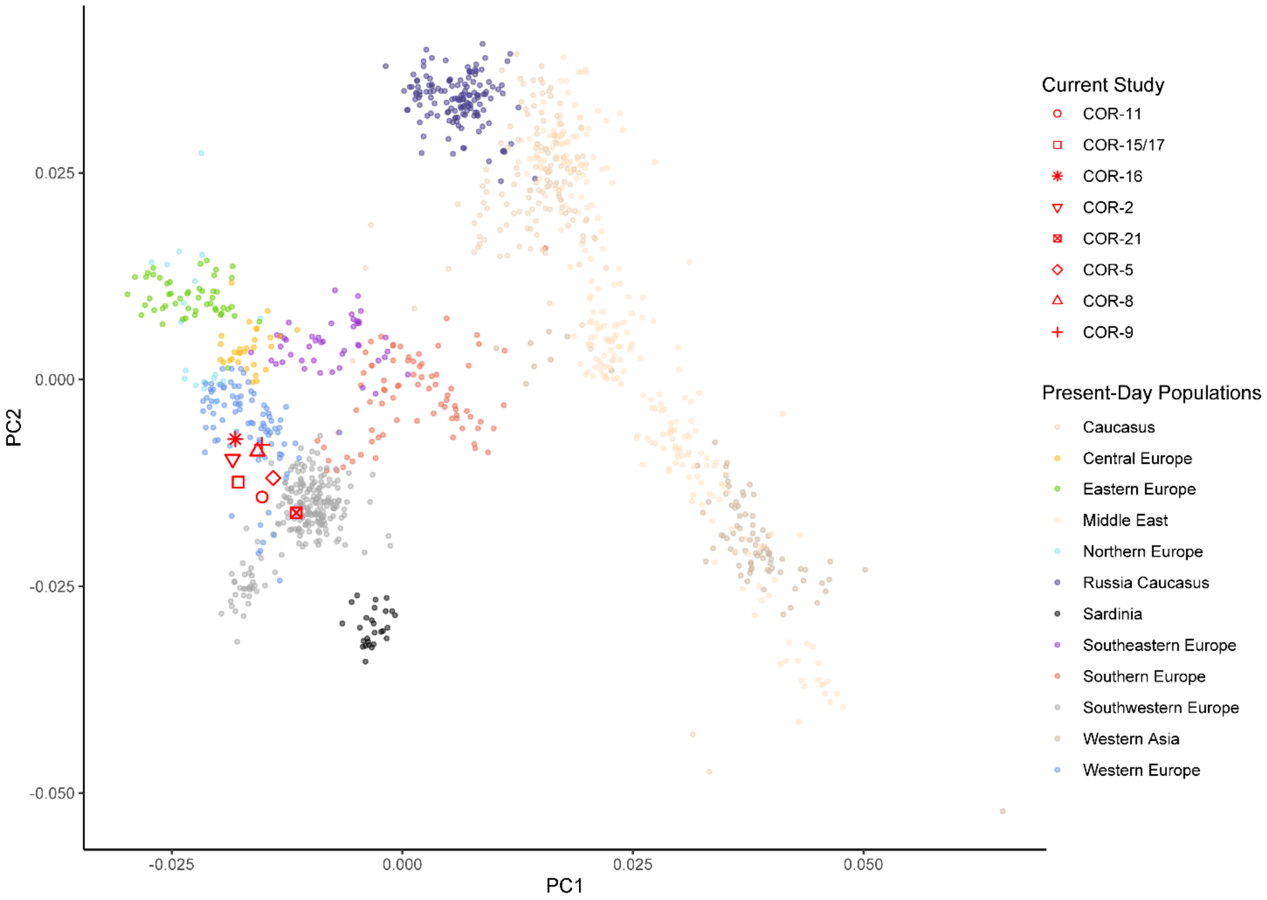

Figure S13. Principal component analysis of ancient samples from Cornaux projected onto the genetic complexity of present-day populations of western Eurasia. Red symbols are used for ancient individuals from Cornaux whereas circles are used for present-day individuals. Different colors indicate the geographic origin.

5. Supplementary references

1. Jud, P. Latènezeitliche Brücken und Strassen der Westschweiz. . In: *Fernkontakte in der Eisenzeit-Konferenz Liblice 2000* (eds. Lang, A., & Salac, V.). Archäologisches Institut der Akademie der Wissenschaften (2002).

2. Pillonel, D., & Reginelli Servais, G. Eisenzeitliche Pfahljochbrücken über die Zihl und die Broye (Schweiz). Zeichnerische Dokumentation und Bauweisen. In: *Archäologie der Brücken, Vorgeschichte – Antike – Mittelalter – Neuzeit / Archaeology of Bridges, Prehistory – Antiquity – Middle Ages – Modern Area*. Pustet Verlag (Bayerische Gesellschaft für Unterwasserarchäologie e. V., in Verbindung mit dem Bayerischen. Landesamt für Denkmalpflege) (2011).

3. Gassmann, P. Nouvelle approche concernant les datations dendrochronologiques du site éponyme de La Tène (Marin-Epagnier, Suisse). *Annuaire d'Archéologie Suisse* **90**, 75-88. (2007).

4. Ramseyer, D. Le pont celtique de Cornaux/Les Sauges: accident ou lieu de sacrifices? In: *Le site de la Tène: bilan des connaissances-état de la question*. Actes de la Table ronde internationale de Neuchâtel, 1-3 Novembre 2007 (eds. Honegger, M., Ramseyer, D., Kaenel, G., Arnold B., & Kaeser, MA.). Office et musée cantonale d'archéologie (Archéologie neuchâteloise), pp. 103-111 (2009).

5. Schwab, H. *Archéologie de la 2e correction des eaux du Jura*. Editions Universitaires Fribourg Suisse (1989).

6. Imhof, U. Die Tiernknochenfunde bei der keltischen Brücke von Cornaux/Les Sauges. In: *Archéologie de la 2e correction des eaux du Jura Vol.1-Les Celtes sur la Broye et la Thielle*. Editions Universitaires Fribourg Suisse, pp. 161-174 (1989).

7. Schwab, H. Entdeckung einer keltischen Brücke an der Zihl und ihre Bedeutung für La Tène. Archäologischer Korrespondenzblatt 2, 289-294 (1972).

8. Lüscher, G., & Müller, F. Sépultures et religion. In: *La Suisse du Paléolithique au Moyen Âge. De l'homme de Néandertal à Charlemagne, 4. Âge du Fer.* (ed. Müller, F.). Société suisse de préhistoire et d'archéologie (1999).

9. Müller, F. Der Massenfund von der Tiefenau bei Bern. Zur Deutung latènezeitlicher Sammelfunde mit Waffen. (1990).

10. Müller, F. *Götter, Gaben, Rituale. Religion in der Frühgeschichte Europas.*Verlag Philipp von Zabern (Kulturgeschichte der antiken Welt, Band 92). (2002).

11. Wyss, R., Rey, T., & Müller, F. *Gewässerfunde aus Port und Umgebung. Katalog der Latène- und römerzeitlichen Funde aus der Zihl.* . Bernisches Historisches Museum. (2002).

12. Kaeser, M.A. La Tène, de la découverte du site à l'éponymie du second âge du Fer européen. Les prospections de Friedrich Schwab et les recherches archéologiques antérieures à la Correction des Eaux du Jura. In: *La Tène: La collection du Musée Schwab (Bienne, Suisse).* (ed. Lejars, T.) (2013).

13. Kaenel, G., & Reginelli Servais, G. La reprise des études du site de La Tène: bilan intermédiaire. *Annuaire d'Archéologie Suisse* **94**, 215-221. (2011).

14. Kaeser, M.A. *La Tène, a Place of Memory. At the origins of Celtic archaeology*. . Laténium. (2022).

15. Garcia, J.P., & Petit, C. Un événement hydrodynamique de haute énergie sur le lac de Neuchâtel pour expliquer le gisement du pont de Cornaux/Les Sauges (et celui de La Tène)? In: *Le site de la Tène: bilan des connaissances-état de la question*. Actes de la Table ronde internationale de Neuchâtel, 1-3 Novembre 2007. (eds. Honegger, M., Ramseyer, D., Kaenel, G., Arnold, B., & Kaeser, MA.). Office et musée d'archéologie, (2009).

16. Müller, F. Beutegut, Opfergaben und Trophäen bei den antiken Kelten. In: *Akten des Vierten Symposiums deutschsprachiger Keltologinnen und Keltologen. Philologische – Historische – Archäologische Evidenzen.*17.-21. Juni 2005 (ed. Birkhan, H.) (2007).

17. Sauter, M.R. Description anthropologique des restes squelettiques humains du pont de Cornaux/Les Sauges. In: *Archéologie de la 2e correction des eaux du Jura Vol.1-Les Celtes sur la Broye et la Thielle*. Editions Universitaires Fribourg Suisse, pp. 137-146 (1989).

18. Schoeninger, MJ, Moore, K. Bone stable isotope studies in archaeology. *J. World Prehist*. **6(2)**, 247–296 (1992). https://doi.org/10.1007/Bf00975551

19. Van der Merwe, N. J. Carbon isotopes, photosynthesis, and archaeology: Different pathways of photosynthesis cause characteristic changes in carbon isotope ratios that make possible the study of prehistoric human diets. *Am. Sci.* **70(6)**, 596–606 (1982).

20. Nehlich, O. The application of Sulphur isotope analyses in archaeological research: A review. *Earth. Sci. Rev*. **142**, 1–17 (2015). https://doi.org/10.1016/j.earscirev.2014.12.0

21. Milella, M. et al. Dining in Tuva: Social correlates of diet and mobility in Southern Siberia during the 2nd–4th centuries CE. *Am. J. Biol. Anthropol.* **178(1)**, 124–139 (2022).https://doi.org/10.1002/ajpa.24506

22. Ambrose, S.H. Preparation and characterization of bone and tooth collagen for isotopic analysis. *J. Archaeol. Sci.* **17**, 431-451 (1990).

23. Ambrose, S.H. Isotopic analysis of paleodiets: methodological and interpretive considerations. In: *Investigations of Ancient Human Tissue: Chemical Analyses in Anthropology* (ed. Sandford, M.K.). Gordon and Breach (1993).

24. DeNiro, M.J. Postmortem preservation and alteration of in vivo bone collagen isotope ratios in relation to palaeodietary reconstruction. *Nature* **317**, 806-809 (1985).

25. Longin, R. New method of collagen extraction for radiocarbon dating. *Nature* **230**, 241-242 (1971).

26. van Klinken, G.J. Bone Collagen Quality Indicators for Palaeodietary and Radiocarbon Measurements. *J. Archaeol. Sci.* **26**, 687-695 (1999).

27. Nehlich, O., & Richards, M.P. Establishing collagen quality criteria for sulfur isotope analysis of archaeological bone collagen. *Archaeol. Anthropol. Sci.* **1**, 59-75 (2009).

28. Salata, G.G., Roelke, L.A. & Cifuentes, L.A. A rapid and precise method for measuring stable carbon isotope ratios of dissolved inorganic carbon. *Mar. Chem*. **69**, 153–161 (2000).

29. Van Geldern, R. et al. Stable carbon isotope analysis of dissolved inorganic carbon (DIC) and dissolved organic carbon (DOC) in natural waters - Results from a worldwide proficiency test. *Rapid. Commun. Mass. Spectrom.* **27**, 2099-2107 (2013).

30. Szidat, S., Vogel, E., Gubler, R., & Lösch, S. Radiocarbon Dating of Bones at the LARA Laboratory in Bern, Switzerland. *Radiocarbon* **59**, 831-842 (2017).

31. Steuri, N. et al. First Radiocarbon Dating of Neolithic Stone Cist Graves from the Aosta Valley (Italy): Insights into the Chronology and Burial Rites of the Western Alpine Region. *Radiocarbon* **65**, 521-538 (2023).

32. Szidat, S. et al. 14C Analysis and Sample Preparation at the New Bern Laboratory for the Analysis of Radiocarbon with AMS (LARA). *Radiocarbon* **56**, 561-566 (2014).

33. Salehpour, M., Håkansson, K., Possnert, G.,Wacker, L., & Synal, H.A. Performance report for the low energy compact radiocarbon accelerator mass spectrometer at Uppsala University. *Nucl. Instrum. Methods. Phys. Res. B.* **371**, 360-364 (2016).

34. Pederzani, S.,& Britton, K. Oxygen isotopes in bioarchaeology: Principles and applications, challenges and opportunities. *Earth. Sci. Rev.* **188**, 77-107 (2019).

35. Britton, K., Fuller, B.T., Tutken, T., Mays, S., & Richards, M.P. Oxygen isotope analysis of human bone phosphate evidences weaning age in archaeological populations. *Am. J. Phys. Anthropol.* **157**, 226-241 (2015).

36. Lightfoot, E.,& O'Connell, T.C. On the Use of Biomineral Oxygen Isotope Data to Identify Human Migrants in the Archaeological Record: Intra-Sample Variation, Statistical Methods and Geographical Considerations. *PLoS One* **11**, e0153850 (2016).

37. Rozanski, K., Araguas-Araguas, L., & Gonfiantini, R. . Isotopic patterns in modern global precipitation. *Geophys. Monogr. Ser.* **78**, 1– 36. (1993).

38. Lee-Thorp, J.A., & van der Merwe, N.J. Carbon isotope analysis of fossil bone apatite. *S. Afr. J. Sci.* **83**, 712-715 (1987).

39. Balasse, M., Ambrose, S.H., Smith, A.B.,& Price, T.D. The Seasonal Mobility Model for Prehistoric Herders in the South-western Cape of South Africa Assessed by Isotopic Analysis of Sheep Tooth Enamel. *J. Archaeol. Sci* **29**, 917-932 (2002).

40. Koch, P.L., Tuross, N., & Fogel, M.L. The Effects of Sample Treatment and Diagenesis on the Isotopic Integrity of Carbonate in Biogenic Hydroxylapatite. *J. Archaeol. Sci.* **24**, 417-429 (1997).

41. Tornero, C., Bălăşescu, A., Ughetto-Monfrin, J., Voinea, V., & Balasse, M. Seasonality and season of birth in early Eneolithic sheep from Cheia (Romania): methodological advances and implications for animal economy. *J. Archaeol. Sci.* **40**, 4039-4055 (2013).

42. Chenery, C.A., Pashley, V., Lamb, A.L, Sloane, H.J., & Evans, J.A. The oxygen isotope relationship between the phosphate and structural carbonate fractions of human bioapatite. *Rapid. Commun. Mass. Spectrom.* **26**, 309-319 (2012).

43. Hyndman, R.J., Einbeck, J., & Wand, M.P. _hdrcde: Highest Density Regions and Conditional Density Estimation_. R package version 3.4 (2021) <https://pkg.robjhyndman.com/hdrcde/>.

44. Sheather, S.J., & Jones, M.C. A Reliable Data-Based Bandwidth Selection Method for Kernel Density Estimation. *J. R. Statist. Soc. B.* **53(3)**, 683–690 (1991).

45. Price, T.D., Burton, J.H., & Bentley, RA. The Characterization of Biologically Available Strontium Isotope Ratios for the Study of Prehistoric Migration. *Archaeometry* **44**, 117-135 (2002).

46. Strauss, A. et al. The Oldest Case of Decapitation in the New World (Lapa do Santo, East-Central Brazil). *PLoS One* **10**, e0137456 (2015).

47. Humphrey, L.T., Dean, M.C., Jeffries, T.E., & Penn, M. Unlocking evidence of early diet from tooth enamel. *Proc. Natl. Acad. Sci. USA.* **105**, 6834-6839 (2008).

48. Bentley, R. Strontium Isotopes from the Earth to the Archaeological Skeleton: A Review. *J. Archaeol. Method. Theory.* **13**, 135-187 (2006).

49. Ericson, J.E. Strontium isotope characterization in the study of prehistoric human ecology. *J. Hum. Evol.* **14**, 503-514 (1985).

50. Faure, G., & Powell, J.L. *Strontium Isotope Geology* (1972).

51. Copeland, S.R. et al. Strontium isotope investigation of ungulate movement patterns on the Pleistocene Paleo-Agulhas Plain of the Greater Cape Floristic Region, South Africa. *Quat. Sci. Rev.* **141**, 65-84 (2016)

52. Wong, M. et al. A bioavailable baseline strontium isotope map of southwestern Turkey for mobility studies. *J. Archaeol. Sci. Rep.* **37,** 102922 (2021).

53. Pin, C., Briot, D., Bassin, C., & Poitrasson, F. Concomitant separation of strontium and samarium-neodymium for isotopic analysis in silicate samples, based on specific extraction chromatography. *Anal. Chim. Acta.* **298**, 209-217 (1994).

54. Damgaard, P.B. et al. Improving access to endogenous DNA in ancient bones and teeth. *Sci Rep* **5**, 11184 (2015).

55. Rohland, N., Siedel, H. & Hofreiter, M. A rapid column-based ancient DNA extraction method for increased sample throughput. *Mol Ecol Resour* **10**, 677-683 (2010).

56. QiaGen. Sample to Insight__ MinElute ® Handbook MinElute PCR Purification Kit. https://www.qiagen.com/cn/resources/download.aspx?id=8f6b09b2-6dcd-4b55-bb4a-255ede40ca3b&lang=en (2020).

57. Promega. QuantusTM Fluorometer Instructions for Use of Product E6150. www.promega.com (2022).

58. Meyer M, Kircher M. Illumina sequencing library preparation for highly multiplexed target capture and sequencing. *Cold Spring Harb Protoc* **2010**, pdb prot5448 (2010).

59. Agilent Technologies. Agilent Technologies Agilent 2100 Bioanalyzer System 2100 Expert Software User's Guide. https://www.agilent.com/cs/library/usermanuals/public/2100_Bioanalyzer_Expert_USR.pdf (2020).

60. Twist Bioscience. Twist Target Enrichment Standard Hybridization v1 Protocol For use with the Twist NGS Workflow. https://www.twistbioscience.com/resources/protocol/twist-target-enrichment-standard-hybridization-v1-protocol (2022).

61. Rohland, N. et al. Three assays for in-solution enrichment of ancient human DNA at more than a million SNPs. *Genome Res* **32**, 2068-2078 (2022).

62. Zhang, J., Kobert, K., Flouri, T. & Stamatakis, A. PEAR: a fast and accurate Illumina Paired-End reAd mergeR. *Bioinformatics* **30**, 614-620 (2014).

63. Li, H. & Durbin, R. Fast and accurate long-read alignment with Burrows-Wheeler transform. *Bioinformatics* **26**, 589-595 (2010).

64. Peltzer, A. et al. EAGER: efficient ancient genome reconstruction. *Genome Biol* **17**, 60 (2016).

65. Jonsson, H., Ginolhac, A., Schubert, M., Johnson, P.L. & Orlando, L. mapDamage2.0: fast approximate Bayesian estimates of ancient DNA damage parameters. *Bioinformatics* **29**, 1682-1684 (2013).

66. Renaud, G., Slon, V., Duggan, A.T. & Kelso, J. Schmutzi: estimation of contamination and endogenous mitochondrial consensus calling for ancient DNA. *Genome Biol* **16**, 224 (2015).

67. Korneliussen, T.S., Albrechtsen, A. & Nielsen, R. ANGSD: Analysis of Next Generation Sequencing Data. *BMC Bioinformatics* **15**, 356 (2014).

68. Skoglund, P., et al. Separating endogenous ancient DNA from modern day contamination in a Siberian Neandertal. *Proc Natl Acad Sci U S A* **111**, 2229-2234 (2014).

69. Skoglund, P., Storå, J., Götherström, A. & Jakobsson, M. Accurate sex identification of ancient human remains using DNA shotgun sequencing. *Journal of Archaeological Science* **40**, 4477-4482 (2013).

70. Mittnik, A., Wang, C.C., Svoboda, J. & Krause, J. A Molecular Approach to the Sexing of the Triple Burial at the Upper Paleolithic Site of Dolni Vestonice. *PLoS One* **11**, e0163019 (2016).

71. Schönherr, S., Weissensteiner, H., Kronenberg, F. & Forer, L. Haplogrep 3 - an interactive haplogroup classification and analysis platform. *Nucleic Acids Res.* **51**,W263-W268 (2023)

72. Ralf, A., Montiel Gonzalez, D., Zhong, K. & Kayser, M. Yleaf: Software for Human Y-Chromosomal Haplogroup Inference from Next-Generation Sequencing Data. *Mol Biol Evol* **35**, 1291-1294 (2018).

73. Monroy Kuhn, J.M., Jakobsson, M. & Gunther, T. Estimating genetic kin relationships in prehistoric populations. *PLoS One* **13**, e0195491 (2018).

74. Fernandes, D.M., Cheronet, O., Gelabert, P. & Pinhasi, R. TKGWV2: an ancient DNA relatedness pipeline for ultra-low coverage whole genome shotgun data. *Sci Rep* **11**, 21262 (2021).

75. Popli, D., Peyregne & S., Peter, B.M. KIN: a method to infer relatedness from low-coverage ancient DNA. *Genome Biol* **24**, 10 (2023).

76. Li, H. et al. The Sequence Alignment/Map format and SAMtools. *Bioinformatics* **25**, 2078-2079 (2009).

77. Mallick, S. et al. The Allen Ancient DNA Resource (AADR): A curated compendium of ancient human genomes. bioRxiv [Preprint].04.06.535797 (2023).

78. Allentoft, M.E. et al. Population genomics of Bronze Age Eurasia. *Nature* **522**, 167-172 (2015).

79. Aneli, S. et al. The Genetic Origin of Daunians and the Pan-Mediterranean Southern Italian Iron Age Context. *Mol Biol Evol* **39**, (2022).

80. Antonio, M.L. et al. Ancient Rome: A genetic crossroads of Europe and the Mediterranean. *Science* **366**, 708-714 (2019).

81. Antonio, M.L. et al. Stable population structure in Europe since the Iron Age, despite high mobility. *eLife* **13**:e79714 (2024).

82. Broushaki, F., et al. Early Neolithic genomes from the eastern Fertile Crescent. *Science* **353**, 499-503 (2016).

83. Brunel, S. et al. Ancient genomes from present-day France unveil 7,000 years of its demographic history. *Proc Natl Acad Sci U S A* **117**, 12791-12798 (2020).

84. Feldman, M. et al. Late Pleistocene human genome suggests a local origin for the first farmers of central Anatolia. *Nat Commun* **10**, 1218 (2019).

85. Fernandes, D.M. et al. The spread of steppe and Iranian-related ancestry in the islands of the western Mediterranean. *Nat Ecol Evol* **4**, 334-345 (2020).

86. Fu, Q. et al. The genetic history of Ice Age Europe. *Nature* **534**, 200-205 (2016).

87. Furtwangler, A. et al. Ancient genomes reveal social and genetic structure of Late Neolithic Switzerland. *Nat Commun* **11**, 1915 (2020).

88. Gnecchi-Ruscone, G.A. et al. Ancient genomic time transect from the Central Asian Steppe unravels the history of the Scythians. *Sci Adv* **7**, (2021).

89. Gokhman, D. et al. Differential DNA methylation of vocal and facial anatomy genes in modern humans. *Nat Commun* **11**, 1189 (2020).

90. Gonzalez-Fortes, G. et al. Paleogenomic Evidence for Multi-generational Mixing between Neolithic Farmers and Mesolithic Hunter-Gatherers in the Lower Danube Basin. *Curr Biol* **27**, 1801-1810 e1810 (2017).

91. Haber, M. et al. A Genetic History of the Near East from an aDNA Time Course Sampling Eight Points in the Past 4,000 Years. *Am J Hum Genet* **107**, 149-157 (2020).

92. Harney, E. et al. A minimally destructive protocol for DNA extraction from ancient teeth. *Genome Res* **31**, 472-483 (2021).

93. Hofmanova, Z. et al. Early farmers from across Europe directly descended from Neolithic Aegeans. *Proc Natl Acad Sci U S A* **113**, 6886-6891 (2016).

94. Jones, E.R. et al. Upper Palaeolithic genomes reveal deep roots of modern Eurasians. *Nat Commun* **6**, 8912 (2015).

95. Krzewinska, M. et al. Ancient genomes suggest the eastern Pontic-Caspian steppe as the source of western Iron Age nomads. *Sci Adv* **4**, eaat4457 (2018).

96. Lazaridis, I. et al. The genetic history of the Southern Arc: A bridge between West Asia and Europe. *Science* **377**, eabm4247 (2022).

97. Lazaridis, I. et al. Genomic insights into the origin of farming in the ancient Near East. *Nature* **536**, 419-424 (2016).

98. Lazaridis, I. et al. Ancient human genomes suggest three ancestral populations for present-day Europeans. *Nature* **513**, 409-413 (2014).

99. Marcus, J.H. et al. Genetic history from the Middle Neolithic to present on the Mediterranean island of Sardinia. *Nat Commun* **11**, 939 (2020).

100. Mathieson, I. et al. The genomic history of southeastern Europe. *Nature* **555**, 197-203 (2018).

101. Mathieson, I. et al. Genome-wide patterns of selection in 230 ancient Eurasians. *Nature* **528**, 499-503 (2015).

102. Moots, H.M. et al. A genetic history of continuity and mobility in the Iron Age central Mediterranean. *Nat Ecol Evol* **7**, 1515-1524 (2023).

103. Narasimhan, V.M. et al. The formation of human populations in South and Central Asia. *Science* **365**, (2019).

104. Olalde, I. et al. Derived immune and ancestral pigmentation alleles in a 7,000-year-old Mesolithic European. *Nature* **507**, 225-228 (2014).

105. Olalde, I. et al. The genomic history of the Iberian Peninsula over the past 8000 years. *Science* **363**, 1230-1234 (2019).

106. Patterson, N. et al. Large-scale migration into Britain during the Middle to Late Bronze Age. *Nature* **601**, 588-594 (2022).

107. Posth, C. et al. The origin and legacy of the Etruscans through a 2000-year archeogenomic time transect. *Sci Adv* **7**, eabi7673 (2021).

108. Schiffels, S. et al. Iron Age and Anglo-Saxon genomes from East England reveal British migration history. *Nat Commun* **7**, 10408 (2016).

109. Unterlander, M. et al. Ancestry and demography and descendants of Iron Age nomads of the Eurasian Steppe. *Nat Commun* **8**, 14615 (2017).

110. Yu, H. et al. Genomic and dietary discontinuities during the Mesolithic and Neolithic in Sicily. *iScience* **25**, 104244 (2022).

111. Haak, W. et al., Massive migration from the steppe was a source for Indo-European languages in Europe. *Nature* **522**, 207–211 (2015).

112. Price, A.L. et al. Principal components analysis corrects for stratification in genome-wide association studies. *Nat Genet* **38**, 904-909 (2006).
